# Caspase 8 deletion causes infection/inflammation-induced bone marrow failure and MDS-like disease in mice

**DOI:** 10.1038/s41419-024-06660-3

**Published:** 2024-04-18

**Authors:** Shanhui Liu, Kanak Joshi, Lei Zhang, Wenyan Li, Ryan Mack, Austin Runde, Patrick A. Hagen, Kevin Barton, Peter Breslin, Hong-Long Ji, Ameet R. Kini, Zhiping Wang, Jiwang Zhang

**Affiliations:** 1 Oncology Institute, Cardinal Bernardin Cancer Canter, Loyola University Chicago Medical Center, Maywood, IL 60153 USA; 2 Department of Cancer Biology, Loyola University Chicago Medical Center, Maywood, IL 60153 USA; 3 Lanzhou University Second Hospital, Key Laboratory of Urological Diseases in Gansu Province, Lanzhou, Gansu 730030 China; 4 Cyrus Tang Hematology Center, Collaborative Innovation Center of Hematology, National Clinical Research Center for Hematologic Diseases, Soochow University, Suzhou, 215123 China; 5 Department of Medicine, Loyola University Chicago Medical Center, Maywood, IL 60153 USA; 6 Departments of Biology and Molecular/Cellular Physiology, Loyola University Chicago, Maywood, IL 60153 USA; 7 Department of Surgery, Loyola University Chicago Medical Center, Maywood, IL 60153 USA; 8 Departments of Pathology and Radiation Oncology, Loyola University Chicago Medical Center, Maywood, IL 60153 USA

**Keywords:** Experimental models of disease, Myelodysplastic syndrome

## Abstract

Myelodysplastic syndromes (MDS) are a heterogeneous group of pre-leukemic hematopoietic disorders characterized by cytopenia in peripheral blood due to ineffective hematopoiesis and normo- or hypercellularity and morphologic dysplasia in bone marrow (BM). An inflammatory BM microenvironment and programmed cell death of hematopoietic stem/progenitor cells (HSPCs) are thought to be the major causes of ineffective hematopoiesis in MDS. Pyroptosis, apoptosis and necroptosis (collectively, PANoptosis) are observed in BM tissues of MDS patients, suggesting an important role of PANoptosis in MDS pathogenesis. Caspase 8 (Casp8) is a master regulator of PANoptosis, which is downregulated in HSPCs from most MDS patients and abnormally spliced in HSPCs from MDS patients with *SRSF2* mutation. To study the role of PANoptosis in hematopoiesis, we generated inducible Casp8 knockout mice (*Casp8*^*−/−*^). *Mx1-Cre-Casp8*^*−/−*^ mice died of BM failure within 10 days of polyI:C injections due to depletion of HSPCs. *Rosa-ERT2Cre-Casp8*^*−/−*^ mice are healthy without significant changes in BM hematopoiesis within the first 1.5 months after *Casp8* deletion. Such mice developed BM failure upon infection or low dose polyI:C/LPS injections due to the hypersensitivity of *Casp8*^*−/−*^ HSPCs to infection or inflammation-induced necroptosis which can be prevented by *Ripk3* deletion. However, impaired self-renewal capacity of *Casp8*^*−/−*^ HSPCs cannot be rescued by *Ripk3* deletion due to activation of Ripk1-Tbk1 signaling. Most importantly, mice transplanted with *Casp8*^*−/−*^ BM cells developed MDS-like disease within 4 months of transplantation as demonstrated by anemia, thrombocytopenia and myelodysplasia. Our study suggests an essential role for a balance in Casp8, Ripk3-Mlkl and Ripk1-Tbk1 activities in the regulation of survival and self-renewal of HSPCs, the disruption of which induces inflammation and BM failure, resulting in MDS-like disease.

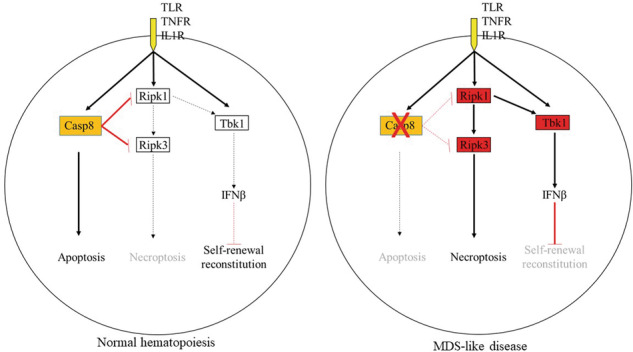

## Facts

- *Casp8*^*−/−*^ mice develop BM failure under infectious and inflammatory conditions, which can be prevented by *Ripk3* deletion;
- *Casp8*^*−/−*^ HSCs exhibit impaired self-renewal which cannot be rescued by *Ripk3* deletion;
- Mice that received *Casp8*^*−/−*^ BM cells developed MDS like-disease at 4 months post-transplantation.

## Introduction

Myelodysplastic syndromes (MDS) are a heterogeneous group of pre-leukemic hematopoietic disorders characterized by persistent cytopenia in peripheral blood (PB) and normo- or hypercellularity and morphologic dysplasia in BM. Ineffective hematopoiesis has been identified as the mechanism leading to the cytopenia, while increased genetic instability and the accumulation of additional mutations are now known to be the mechanism explaining the elevated risk for leukemic transformation in MDS patients [1, 2]. An inflammatory BM micro-environment and programmed cell death (PCD) of hematopoietic stem/progenitor cells (HSPCs) have been thought to be the major causes leading to ineffective hematopoiesis [3–6]. Three types of PCD, apoptosis, necroptosis and pyroptosis were observed in BM tissues of MDS patients, suggesting PANoptosis [7–9]. Caspase 8 (Casp8) is a master regulator of PANoptosis, which is downregulated or abnormally spliced in HSPCs from patients with MDS [4, 10].

Casp8 was first identified as a key mediator of the extrinsic apoptotic pathway triggered by death receptors including Fas, Trail-receptors, tumor necrosis factor receptor (TNFR), and DR3 [11], as well as Toll-like receptors (TLRs) [12]. Upon ligand binding, death receptors and TLRs induce the activation of Casp8-apoptosis by triggering the formation of a death-inducing signaling complex (Fadd/pro-Casp8) for Fas and Trail-receptors [13, 14], or a complex IIa (Ripk1/Fadd/pro-Casp8) for TNFR1, DR3 and TLRs [12, 13, 15]. Under Casp8-inactive conditions, death receptors and TLRs induce necroptosis by triggering the formation of a necrosome or complex IIb (Ripk3/Ripk1/Fadd/pro-Casp8) [16]. Recent studies demonstrated that pro-Casp8 also interacts with ASC (the adaptor molecule, apoptosis-associated speck-like protein containing a CARD) to regulate Nlpr3-inflammasome activity and pyroptosis [17–19]. Detailed studies demonstrated that TLR signaling actually induces a much bigger complex called a PANoptosome which contains almost all the key components of the above three complexes [7, 20]. This complex of proteins mediates PANoptosis. Tak1 (TGF-β-activated kinase 1) [9, 21, 22], Zbp1 (Z-DNA binding protein 1) [9, 23], Ripk1 [24] and IRF1 [25] are the master regulators of PANoptosis, while Casp8 plays a key molecular role in regulating the switching among the three types of PCD [26, 27].

The aspartate-specific proteolytic activity of pro-Casp8 is normally inhibited by its N-terminal domains and other regulatory proteins. Activation of such activity allows Casp8 to cleave and activate both itself and its substrates. Activated Casp8 triggers mitochondria-independent apoptosis by directly activating the executioner Caspases including Casps3 and 7 or, in certain circumstances, induces mitochondrial-dependent apoptosis by cleaving Bid to produce pro-apoptotic tBid [28, 29]. Activated Casp8 also forms a noncanonical inflammasome with Card9/Bcl10/MALT1/ASC or Ripk1/ASC/Nlrp3 to cleave IL-1β, IL-18 and GSDMD for IL-1β/IL-18 production and pyroptosis [30, 31]. However, activated Casp8 restricts TNFα and TLR-induced Ripk3/Mlkl-mediated necroptosis by cleaving and degrading Ripk1 and Ripk3 and likely Mlkl as well [32–34]. In addition, Casp8 can also induce cell death through its catalytic-independent scaffolding activity. For example, Casp8 interacts with ASC and activates canonical Nlpr3-Casp1/11-mediated IL-1β production and pyroptosis [26, 35, 36]. Inhibition of Casp8 activity represses apoptosis but enhances necroptosis, while activation of Casp8 represses necroptosis but enhances apoptosis. The role of Casp8 in the regulation of pyroptosis is cell context-dependent [22, 26, 27, 37, 38].

In addition to regulating PCD, Casp8 is also involved in the regulation of cell proliferation/mitosis, differentiation, cytokine production and DNA damage repair. It was found that during mitosis, Ripk1/Casp8/Ripk3 form a mitotic ripoptosome complex together with Polo-like kinase 1 (Plk1). Casp8 regulates the faithful segregation of chromosomes and stability by cleaving Plk1. Inhibition of Ripk1 or Casp8 leads to chromosomal instability [39]. During radiation or chemical-induced DNA damage, Casp8 also forms a DNA damage sensor complex with Fadd/c-FLIP/Ripk1, orchestrating replication-associated DNA damage and H2AX phosphorylation [40]. DNA damage-triggered activation of nuclear Casp8 also overcomes the p53-dependent G2/M checkpoint through the cleavage of USP28, leading to drug-resistance [41]. All these studies suggested that Casp8 is essential for maintaining chromosomal stability and DNA damage repair, independent of its roles in cell death and its inflammatory function [42]. In addition, in responding to TNFα and TLR3/4 stimulation, Casp8 cleaves N4BP1 (NEDD4-binding protein 1), a negative regulator of genes encoding inflammatory cytokines, thus promoting the expression of cytokines, including IL-6, G-CSF and TNFα as well as chemokines such as CXCL1 and CCL3 [43]. Casp8 also regulates inflammatory cytokine production by activating inflammasomes in catalytic activity-dependent or independent processes [26, 27, 44].

To study the role of Casp8 in adult hematopoiesis, Kang et al. analyzed *Mx1Cre*-mediated *Casp8* knockout (*Casp8*^*−/−*^) mice and reported that although there are no changes in the number of phenotypic HSPCs, functional HSPCs are significantly reduced as assessed by their ability to form myeloid or B lymphoid colonies as well as repopulating BM and lymphoid organs [45]. However, all these analyses were conducted shortly after polyI:C injections, and thus cannot distinguish Casp8-intrinsic effects from polyI:C- induced effects. In this study, we comparatively studied the hematopoietic phenotypes and HSPC functions between *Mx1Cre*^*+*^*Casp8*^*fx/fx*^ and *Rosa26-ERTCre*^*+*^*Casp8*^*fx/fx*^ mice as well as *Mx1Cre*^*+*^*Casp8*^*fx/fx*^*Ripk3*^*−/−*^ and *Rosa26-ERTCre*^*+*^*Casp8*^*fx/fx*^*Ripk3*^*−/−*^ mice. We found that *Mx1Cre*^*+*^*Casp8*^*fx/fx*^ mice developed severe BM failure after polyI:C-induced *Casp8* deletion due to the massive necroptosis of HSPCs. This could be prevented by *Ripk3* deletion. Interestingly, the hematopoiesis of *Rosa26-ERTCre*^*+*^*Casp8*^*fx/fx*^ mice 1.5 month after tamoxifen (TAM)-induced *Casp8* deletion is comparable to that in *wild-type* (*WT*) mice. However, such mice developed MDS-like diseases 4 months after Casp8 deletion. Our study demonstrates a critical role for Casp8 in preventing infection and inflammation-induced Ripk3-mediated necroptosis in HSPCs. In addition, Casp8 also regulates HSC self-renewal by restricting Ripk1-Tbk1-mediated Type I interferon (IFN) production.

## Results

### Basal level activation of Casp8 limits Ripk1-Ripk3-Mlkl signaling in c-Kit^+^ HSPCs

**Fig. 1:**
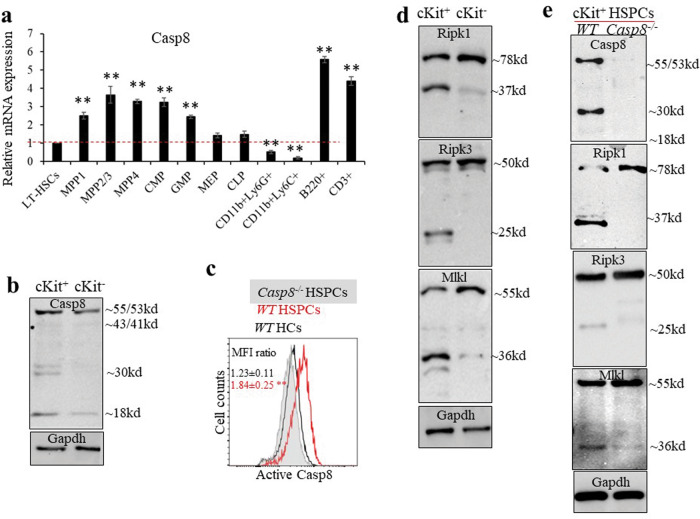
Basal levels of Casp8 activation were detected in cKit^+^ HSPCs. **a** HSCs (LSK-CD135^-^CD150^+^CD48^-^), MPP1s (LSK-CD135^-^CD150^-^CD48^-^), MPP2/3s (LSK-CD135^-^CD150^+/^CD48^+^), MPP4s (LSK-CD135^+^CD150^-^CD48^+/low^), CMPs (LK-CD16/32^-^CD34^+^), GMPs (LK-CD16/32^+^CD34^+^), MEPs (LK-CD16/32^-^CD34^−^), CLPs (Lineage^−^c-Kit^low^Sca1^low^CD127^+^), granulocytes (CD11b^+^Ly6G^+^), monocytes (CD11b^+^Ly6C^+^), B lymphocytes (B220^+^) and T lymphocytes (CD3^+^) were purified from C57Bl6/J mice by FACS. Casp8 expression in all populations was examined by RT-PCR and normalized to the level in HSCs. Triplicate experiments were conducted. Data represent one of the two experiments performed in triplicate. **p < 0.01 compared to HSCs. **b** Basal levels of Casp8 activation were detected in c-Kit^+^ HSPCs but not in c-Kit^-^ HCs as shown by Western blotting. **c** Active-Casp8 levels were examined and compared among LSK HSPCs and c-Kit^-^ HCs from *WT* mice by flow cytometry. MFI (mean fluorescence intensity) ratio was counted from 3 independent experiments and normalized to gene knockout controls. **p < 0.01 compared to *WT* HCs. BM LSK HSPCs isolated from *Casp8*^*−/−*^ mice were studied in parallel as controls. Levels of Ripk1, Ripk3, and Mlkl were compared between c-Kit^+^ HSPCs and c-Kit^-^ HCs from WT mice (**d**) and between c-Kit^+^ HSPCs isolated from *WT* and Casp8^−/−^ mice (**e**) by Western blotting. Data in **b**, **d**, **e** represent one example from the three independent experiments.

Casp8 expression is higher in c-Kit^+^ HSPCs than that in c-Kit^-^ BM hematopoietic cells (HCs) as shown by both RT-PCR (Fig. 1a) and Western blotting (Fig. 1b) assays. Within BM cells, granulocytes and monocytes express relatively low Casp8 (Fig. 1a). Interestingly, basal levels of Casp8 activity were detected in c-Kit^+^ HSPCs but not in c-Kit^-^ HCs as demonstrated by increased number of truncated forms of Casp8 and flow cytometric analysis of active Casp8 staining (Fig. 1b, c). In addition, increased levels of truncated forms of Ripk1, Ripk3 and Mlkl were detected in c-Kit^+^ HSPCs (Fig. 1d). Furthermore, we found that the truncated forms of Ripk1, Ripk3 and Mlkl in c-Kit^+^ HSPCs can be prevented by Casp8 knockout (Fig. 1e). All these data suggest that during normal homeostatic conditions, c-Kit^+^ HSPCs have a basal level activation of Casp8 which restricts Ripk1-Ripk3-Mlkl signaling in c-Kit^+^ HSPCs by cleaving Ripk1, Ripk3 and Mlkl. However, the cleavage of Casp3, Casp7, Casp1, and Casp11 as well as the substrates PARP1 and GSDMD was not detected in c-Kit^+^ HSPCs, suggesting that such levels of Casp8 activation are insufficient to activate apoptosis or pyroptosis (Fig. S1).

### Mx1Cre^+^Casp8^fx/fx^ mice developed BM failure after polyI:C-induced Casp8 deletion, which can be prevented by Ripk3 deletion

**Fig. 2:**
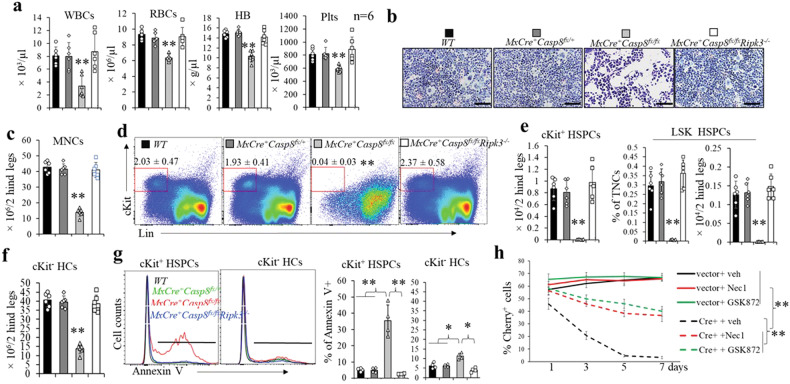
BM failure in polyI:C-induced Casp8^−/−^ mice can be completely prevented by Ripk3 deletion. **a–f** *WT*, *MxCre*^*+*^*Casp8*^*fx/+*^, *MxCre*^*+*^*Casp8*^*fx/fx*^ and *MxCre*^*+*^*Casp8*^*fx/fx*^*Ripk3*^*−/−*^ mice were injected with 5 μg/g body weight polyI:C every other day for a total of three injections. PB and BM were collected from another batch of mice (6 for each genotype) 8 days after the last polyI:C injection. WBCs, RBCs, HB and platelets in PB were examined by a Hemavet 950FS (**a**); BM cellularity was compared by histology (**b**) and MNC counts (**c**), percentages and numbers of c-Kit^+^ HSPCs (**d**, **e**) and LSK HSPCs (**d**, **f**), as well as cKit^-^ HCs, were analyzed by flow cytometry. **g** BM MNCs were collected two days after a single polyI:C injection (5 μg/g body weight). Death of cKit^+^ HSPCs and cKit^-^ HCs was examined by Annexin-V staining. **h** cKit^+^ HSPCs were collected from *Casp8*^*fx/fx*^ mice and infected with either Cherry-expressing virus or Cherry/*Cre*-expressing virus. The infected cells were then cultured in HSPC culture medium (RPMI 1640 supplied with 10% FBS, 100 ng/ml SCF, 50 ng/ml TPO, 10 ng/ml IL3 and 25 ng/ml IL6) with or without 30 μM Nec-s or 3 μM GSK’872 with medium change every other day. The percentages of Cherry^+^ cells were examined by FACS on the indicated days. Triplicate experiments were conducted. In (**a–g**). **p < 0.01 compared to other groups.

We have reported that Ripk3 signaling is not required for homeostatic hematopoiesis [46]. To study the role of Casp8 in the regulation of normal hematopoiesis, we generated *Mx1Cre*^*+*^*Casp8*^*fx/fx*^, *Mx1Cre*^*+*^*Casp8*^*fx/+*^*, Casp8*^*fx/fx*^ (*WT*) and *Mx1Cre*^*+*^*Casp8*^*fx/fx*^*Ripk3*^*−/−*^ mice. At 8 weeks of age, all mice were administered polyI:C every other day for a total of three injections. Eight to ten days after these polyI:C injections, all *Mx1Cre*^*+*^*Casp8*^*fx/fx*^ mice became frail as shown by hunched backs and reduced mobility, and died within 12 days, while *Mx1Cre*^*+*^*Casp8*^*fx/+*^, *WT* and *Mx1Cre*^*+*^*Casp8*^*fx/fx*^*Ripk3*^*−/−*^ mice were observed to be generally normal (Fig. S2). All *Mx1Cre*^*+*^*Casp8*^*fx/fx*^ mice developed severe BM failure as demonstrated by a reduction of white blood cells (WBC), red blood cells (RBCs) and platelets in PB (Fig. 2a), as well as hypocellularity and a reduction in mononuclear cells (MNCs) in BM (Fig. 2b, c). Interestingly, when analyzed 6 days after the last polyI:C injection, we found that c-kit^+^ HSPCs in *Mx1Cre*^*+*^*Casp8*^*fx/fx*^ mice had almost completely been eliminated from BM (Fig. 2d, e), while c-Kit^-^ HC number was only modestly reduced (Fig. 2f). Compared to c-Kit^-^ HCs, Annexin-V staining showed significantly increased cell death in c-Kit^+^ HSPCs in *Mx1Cre*^*+*^*Casp8*^*fx/fx*^ mice, suggesting a critical role for Casp8 in the survival of HSPCs (Fig. 2g). However, all hematopoietic parameters in *Mx1Cre*^*+*^*Casp8*^*fx/+*^ mice were comparable to those in *WT* mice, since one allele of Casp8 is sufficient to maintain the level of Casp8 expression (Fig. S3) and hence the survival of HSPCs. In addition, all hematopoietic parameters in *Mx1Cre*^*+*^*Casp8*^*fx/fx*^*Ripk3*^*−/−*^ mice were also comparable to those in *WT* mice, indicating that the HSPCs in *Mx1Cre*^*+*^*Casp8*^*fx/fx*^ mice died from Ripk3-mediated necroptosis (Fig. 2a–g). This notion was further supported by in vitro studies where induction of *Casp8* deletion by infection of *Casp8*^*fx/fx*^ HSPCs with *Cre*-expressing virus also caused massive cell death. Such cell death could be largely prevented by treatment with the Ripk1-inhibitor Necrostatin-1 or the Ripk3 inhibitor GSK872 (Fig. 2h).

### Rosa26-ERTCre^+^Casp8^fx/fx^ mice exhibited normal hematopoiesis after TAM-induced Casp8 deletion

**Fig. 3:**
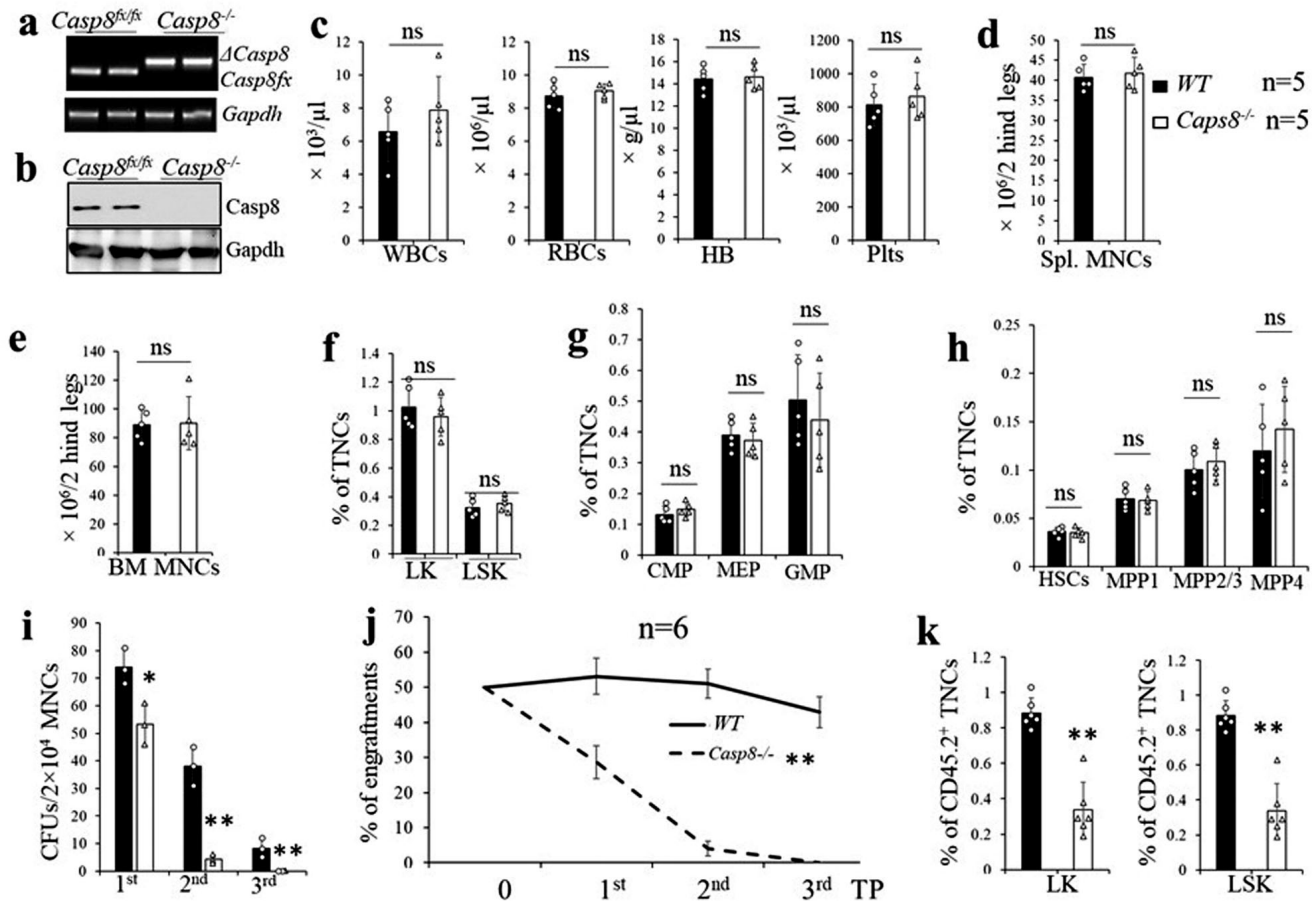
Impaired colony-forming ability and engraftment capacity of Casp8^−/−^ HSPCs. **a–e** *WT* and *Rosa-CreERT*^*+*^*Casp8*^*fx/fx*^ mice were injected with 100 μg/g body weight TAM every day for 5 consecutive days to induce *Casp8* deletion. BM MNCs were collected 30 days after the last TAM injection and efficient *Casp8* deletion was verified by PCR (**a**) and Western blotting (**b**). PB, spleens and BM were collected from *WT* and *Casp8*^*−/−*^ mice 45 days after the last TAM injection; WBCs, RBCs, HB and platelets in PB were examined by a Hemavet 950FS (**c**); spleens and BM cellularity were compared by MNC counts (**d**, **e**); percentages of LSK HSPCs and LK MPs (**f**), percentages of CMP, GMP and MEP (**g**), and percentages of HSCs, MPP1s, MPP2/3s and MPP4s (**h**) in BM of *WT* and *Casp8*^*−/−*^ mice were analyzed by FACS. **i–k** The colony-forming ability of BM MNCs from *WT* and *Casp8*^*−/−*^ mice were compared by serial replating (**i**); the CHRC of *WT* and *Casp8*^*−/−*^ HSCs were assessed by serial transplantation (**j**); the percentages of donor LK MPs and LSK HSPCs from the first transplantation recipient mice were examined 10 wks. post-transplantation (**k**) and represent one of the three independent experiments performed in triplicate. Data in **j**, **k** are a summary of 6 mice per group. **p < 0.01 compared to *WT*.

Because either polyI:C or viral infection can induce necroptosis when Casp8 activity is inhibited, there are two possible mechanisms to explain the BM failure phenotype observed in *Mx1Cre*^*+*^*Casp8*^*fx/fx*^ mice: either *Casp8*^*−/−*^ HSPCs undergo intrinsic necroptosis, or they are sensitive to polyI:C or viral infection-induced necroptosis. To distinguish between these two possible mechanisms, we generated *Rosa26-ERTCre*^*+*^*Casp8*^*fx/fx*^ and *WT* mice. At 8 weeks of age, all mice were injected with TAM daily for 5 days to induce *Casp8* deletion. Complete deletion of *Casp8* in BM HSPCs and MNCs was confirmed by both PCR and Western blot assays in *Rosa26-ERTCre*^*+*^*Casp8*^*fx/fx*^ mice post-TAM injection (*Casp8*^*−/−*^ hereafter) (Fig. 3a, b). In contrast to *Mx1Cre*^*+*^*Casp8*^*fx/fx*^ mice which died from BM failure after polyI:C induction, *Casp8*^*−/−*^ mice are generally normal for two months after TAM injections. Detailed analysis of HCs, including HSPCs (including HSCs, MPP1s, MPP2/3s, MPP4s), and myeloid progenitors (including CMPs, GMPs and MEPs) in BM (Fig. S4), as well as blood cells in PB at 1.5 months post-TAM injections, demonstrated that all hematopoietic parameters in the BM of *Casp8*^*−/−*^ mice were comparable to those of *WT* mice (Fig. 3c–h), suggesting that Casp8 is not essential for the survival of HSPCs during homeostasis. Functional studies demonstrated that BM cells from *Casp8*^*−/−*^ mice displayed a reduced CFU capacity during serial replating (Fig. 3i) and reduced competitive hematopoietic repopulation capacity (CHRC) over serial transplantation when compared to BM cells from *WT* mice (Fig. 3j). Numbers of HSPCs were also reduced during serial transplantation (Fig. 3k), suggesting that Casp8 is required for maintaining the colony-forming capacity of HSPCs and the CHRC of HSCs under stress.

### HSPCs in Casp8^−/−^ mice are more susceptible to infection and polyI:C/LPS-induced cell death

**Fig. 4:**
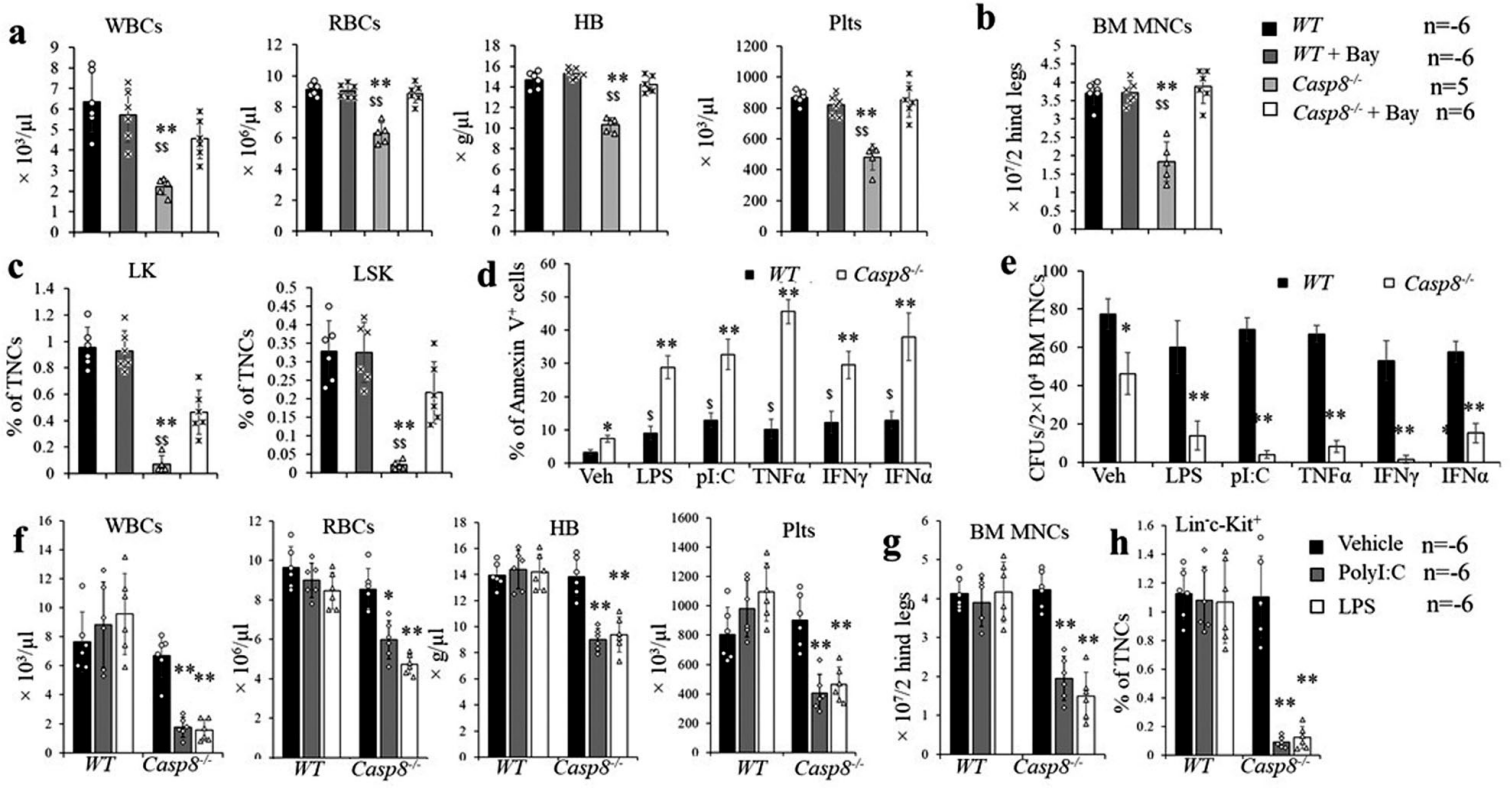
HSPCs in Casp8^−/−^ mice are acutely prone to infection and polyI:C/LPS-induced cell death. **a–c** A batch of *WT* and *Casp8*^*−/−*^ mice (5 days post last TAM injection) was transferred between facilities. Unfortunately, these mice became infected with gram-negative bacteria. All *Casp8*^*−/−*^ mice were frail, as shown by hunched backs and reduced mobility, whereas *WT* mice were generally normal. Half of the mice were randomly selected and given drinking water containing the antibiotic Baytril (enrofloxacin, Bay, 0.16 mg/ml) for twelve days. The PB and BM were collected from all groups of mice. WBCs, RBCs, HB and platelets in PB were examined using a Hemavet 950FS (**a**); BM cellularity was compared by MNC counts (**b**); percentages of LSK HSPCs and LK MPs were analyzed by FACS (**c**). **d**, **e**. cKit^+^ HSPCs were isolated from *WT* and *Casp8*^*−/−*^ mice and cultured in HSPC culture medium with or without LPS (100 ng/ml), polyI:C (10 μg/ml), TNFα (20 ng/ml), IFNγ (20 ng/ml) or IFNα (20 ng/ml) treatment. Cell death was examined by Annexin-V staining 24 h. after culture (**d**); or seeded into methylcellulose medium for colony-forming unit assay with or without LPS (100 ng/ml), polyI:C (10 μg/ml), TNFα (20 ng/ml), IFNγ (20 ng/ml) or IFNα (20 ng/ml) treatment. CFU were counted after 8 days of culturing (**e**). **f–h** *WT* and *Casp8*^*−/−*^ mice were injected with vehicle, polyI:C (1 μg/g every other day), or LPS (0.2 μg/g every other day) for 16 days. PB and BM were collected five days after the last injection. WBCs, RBCs, HB and platelets in PB were analyzed using a Hemavet 950FS (**f**); BM cellularity was compared by MNC counts (**g**); percentages of c-Kit^+^ HSPCs in BM were examined by flow cytometry (**h**). Data in **a–c** and **f–h** are a summary of 5–6 mice in each group. Data in **d**, **e** represent one of the three independent experiments performed in triplicate. * and **p < 0.05 and <0.01, respectively compared to other groups.

During our experiments, we transferred a batch of *Casp8*^*−/−*^ and *WT* mice (five days post-TAM injections) between facilities, after having been first maintained in a quarantine room. We found that all *Casp8*^*−/−*^ mice became sick and developed BM failure within 6-12 days of observation, characterized by pancytopenia (Fig. 4a) and reduced BM cellularity (Fig. 4b) due to significant reduction of HSPCs (Fig. 4c). All *WT* mice at this point were normal (Fig. 4a–c). We speculated that these mice might have bacterial infections, so we treated them with the antibiotic Baytril for 20 days. All the *Casp8*^*−/−*^ mice in the untreated group died of BM failure, while the mice that received Baytril survived and were healthy. Hematopoietic parameters were almost completely restored in the group given Baytril (Fig. 4a–c).

To assess whether the BM failure that developed in *Casp8*^*−/−*^ mice was due to the propensity of *Casp8*^*−/−*^ HSPCs to suffer infections or inflammation-induced cell death, we compared the responses of *Casp8*^*−/−*^ and *WT* HSPCs to low-dose TLR agonists or inflammatory cytokines. We found that polyI:C, LPS, IFNα, IFNγ or TNFα treatment kills significantly more *Casp8*^*−/−*^ HSPCs than *WT* HSPCs (Fig. 4d). In addition, CFU assays demonstrated that polyI:C, LPS, IFNα, IFNγ or TNFα treatment significantly inhibited the colony-forming ability of *Casp8*^*−/−*^ HSPCs but had fewer such effects on *WT* HSPCs (Fig. 4e). Furthermore, low dose polyI:C or LPS injections induced pancytopenia, BM failure and loss of HSPCs in *Casp8*^*−/−*^ mice but did not do so in *WT* mice (Fig. 4f–h).

### Ripk3 deletion prevents polyI:C-induced BM failure in Casp8^−/−^ mice

**Fig. 5:**
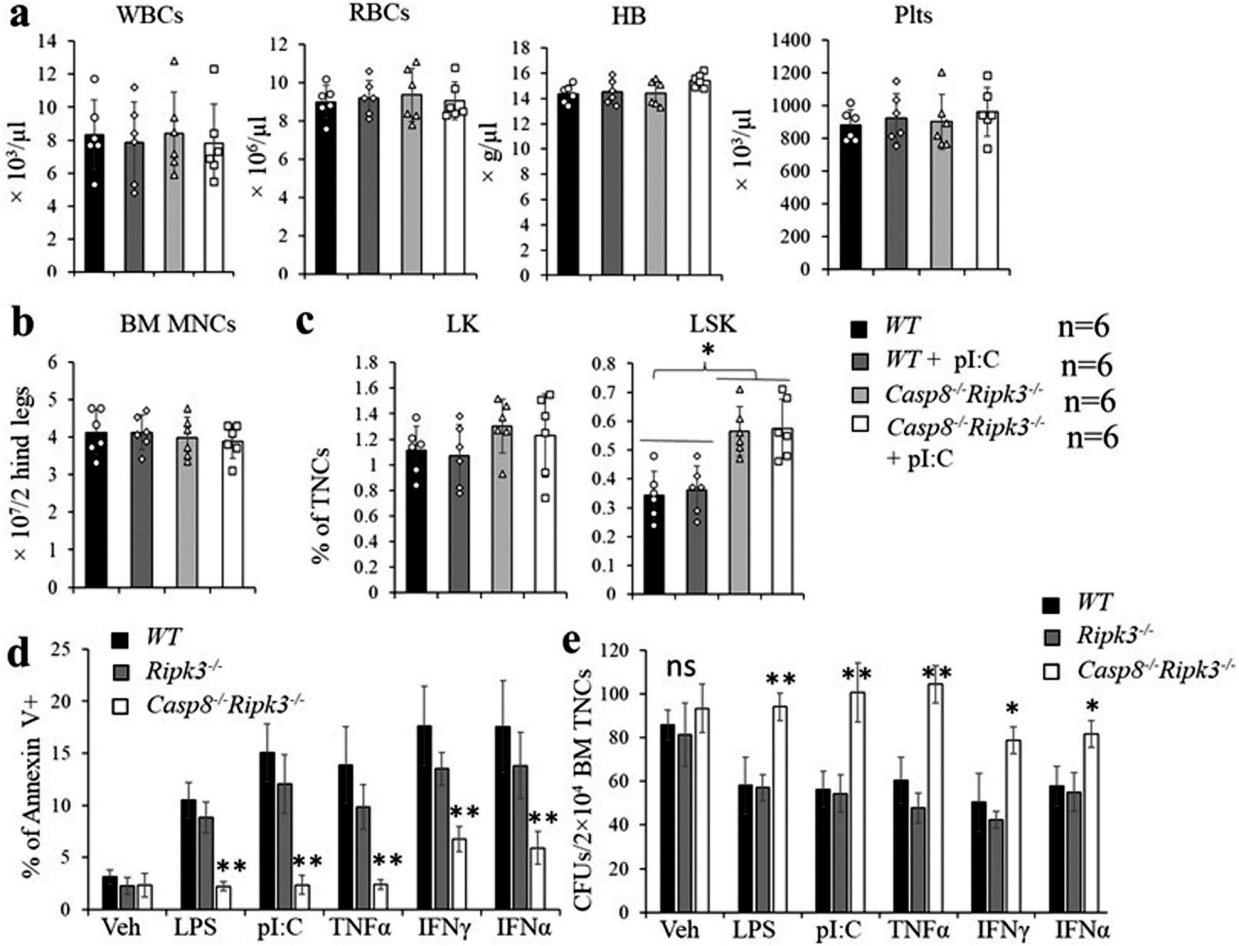
HSPCs in Casp8^−/−^Ripk3^−/−^ mice are resistant to polyI:C/LPS and inflammatory cytokine-induced cell death. **a–c** Batches of *WT* and *Casp8*^*−/−*^*Ripk3*^*−/−*^ mice (10 days post last TAM injection) were injected with vehicle or polyI:C (1 ng/ml every other day) for 16 days; PB and BM were collected 5 days after the last injection. WBCs, RBCs, HB and platelets in PB were determined by a Hemavet 950FS (**a**), BM cellularity was compared by MNC counts (**b**); percentages of LK MPs and LSK HSPCs in BM were examined by flow cytometry (**c**). **d**, **e** cKit^+^ HSPCs were isolated from *WT, Ripk3*^*−/−*^ and *Casp8*^*−/−*^*Ripk3*^*−/−*^ mice and cultured in HSPC culture medium with or without LPS (300 ng/ml), polyI:C (50 μg/ml), TNFα (50 ng/ml), IFNγ (50 ng/ml) or IFNα (50 ng/ml) treatment. Cell death was examined by Annexin-V staining 24 h. after culturing (**d**); or seeded into methylcellulose medium for colony-forming unit forming assay with or without LPS (300 ng/ml), polyI:C (50 μg/ml), TNFα (50 ng/ml), IFNγ (50 ng/ml) or IFNα (50 ng/ml) treatment; CFU were counted on day 8 of culturing (**e**). Data in **a–c** are summary of 6 mice in each group. Data in **d**, **e** represent one of the three independent experiments performed in triplicates. * and **p < 0.05 and <0.01, respectively, compared to other groups.

To study whether TLR agonists or inflammatory cytokines kill *Casp8*^*−/−*^ HSPCs through inducing Ripk3-mediated necroptosis, we generated *Rosa26-ERTCre*^*+*^*Casp8*^*fx/fx*^*Ripk3*^*−/−*^ mice. After TAM induction to induce *Casp8* deletion to generate compound-knockout mice (*Casp8*^*−/−*^*Ripk3*^*−/−*^), we found that *Casp8*^*−/−*^*Ripk3*^*−/−*^ mice were healthy and all hematopoietic parameters in such mice were comparable to those of *WT* mice (Fig. 5a-c). In addition, distinct from *Casp8*^*−/−*^ mice, *Casp8*^*−/−*^*Ripk3*^*−/−*^ mice were resistant to polyI:C-induced BM failure. All hematopoietic parameters in *Casp8*^*−/−*^*Ripk3*^*−/−*^ mice were comparable to those in *WT* mice (Fig. 5a-c). In vitro culture studies demonstrated that, in response to polyI:C, LPS, IFNα, IFNγ or TNFα treatment, the death rate of *Casp8*^*−/−*^*Ripk3*^*−/−*^ HSPCs was significantly lower than that of *WT* and *Ripk3*^*−/−*^ HSPCs (Fig. 5d). In addition, polyI:C, LPS, IFNα, IFNγ or TNFα treatment significantly inhibited the colony-forming capacity of *WT* and *Ripk3*^*−/−*^ HSPCs but had significantly reduced effects on *Casp8*^*−/−*^*Ripk3*^*−/−*^ HSPCs (Fig. 5e). This suggests that *Casp8*^*−/−*^ HSPCs primarily die of necroptosis. This idea is further supported by Mlkl inhibition. GW806742X is a Mlkl inhibitor [47]. We found that the polyI:C, LPS, IFNα, IFNγ or TNFα treatment-induced death of *Casp8*^*−/−*^ HSPCs can also be prevented by GW806742X pre-treatment (Fig. S5).

### Ripk3 deletion fails to fully restore the CHRC of Casp8^−/−^ HSCs

**Fig. 6:**
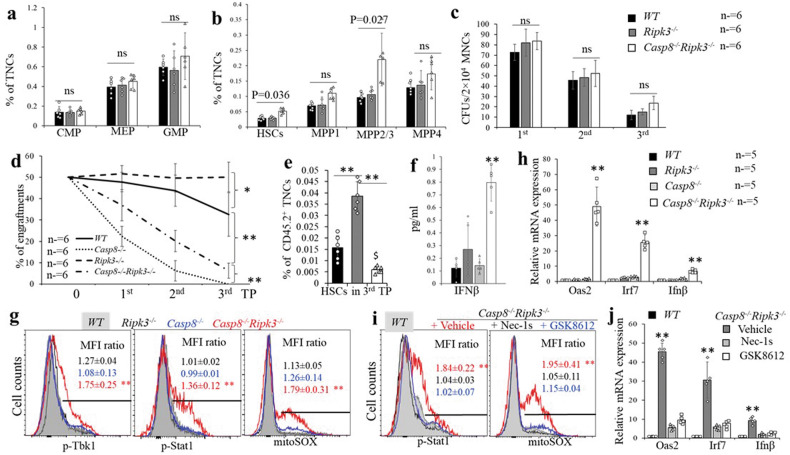
Ripk3 deletion fails to fully restore the CHRC of Casp8^−/−^ HSCs. **a–g** Thirty days after the last TAM injection, BM cells were collected from *WT, Ripk3*^*−/−*^ and *Casp8*^*−/−*^*Ripk3*^*−/−*^ mice and the % of CMPs, GMPs and MEPs (**a**) as well as HSCs, MPP1s, MPP2/3s and MPP4s (**b**) were assessed by flow cytometry. Colony-forming abilities of BM cells were compared by CFU assay (**c**); CHRC was compared by serial transplantation assay and BM cells from *Casp8*^*−/−*^ were studied in parallel as controls (**d**); the % of donor HSC contributions in BM of 3^rd^ round recipient mice were examined by flow cytometry (**e**). Type I IFN concentrations in the BM and sera of *WT, Ripk3*^*−/−*^ and *Casp8*^*−/−*^*Ripk3*^*−/−*^ mice were compared using LEGENDplex™ assay (**f**). cKit^+^ HSPCs were isolated from *WT, Ripk3*^*−/−*^, *Casp8*^*−/−*^ and *Casp8*^*−/−*^*Ripk3*^*−/−*^ mice, pStat1, pTbk1, mito-ROS levels were examined by intracellular antibody or MitoSOX staining (**g**). cKit^+^ HSPCs were collected from *WT, Ripk3*^*−/−*^ and *Casp8*^*−/−*^*Ripk3*^*−/−*^ mice and mRNA expression of the ISG genes was examined by RT-PCR and normalized to the levels in *WT* HSPCs (**h**). **i**, **j** *WT* and *Casp8*^*−/−*^*Ripk3*^*−/−*^ mice were treated with vehicle, 2 μg/g Nes-1s (Ripk1 inhibitor), or 5 μg/g GSK8612 (Tbk1 inhibitor) for 2 days. cKit^+^ HSPCs were isolated from the mice 12 h. post-treatment; pStat1 and mito-ROS levels were examined by intracellular antibody or MitoSOX staining (**i**); the expression of ISG genes was examined by RT-PCR assay (**j**). Data in **a–f** are a summary of 6 mice in each group. Data in **h–j** represents one of the three independent experiments performed in triplicate. * and **p < 0.05 and <0.01, respectively, compared to other groups. MFI ratio in **g** and **i** was counted from 3 independent experiments and normalized to WT controls.

We have reported that Ripk3 is not required for normal homeostatic hematopoiesis but does regulate HSC self-renewal during stress conditions such as serial transplantation [46]. Analysis of phenotypic HSPCs in the BM of *WT*, *Ripk3*^*−/−*^ and *Casp8*^*−/−*^*Ripk3*^*−/−*^ mice showed a significant increase in the LSK cell population in *Casp8*^*−/−*^*Ripk3*^*−/−*^ mice compared to *WT* and *Ripk3*^*−/−*^ mice (Fig. 5c). Detailed analysis suggested that the numbers of HSCs and MPP2/3 cells were increased in *Casp8*^*−/−*^*Ripk3*^*−/−*^ mice compared to *WT* and *Ripk3*^*−/−*^ mice, while the MPP1, MPP4, CMP, MEP and GMP populations were comparable to *WT* and *Ripk3*^*−/−*^ mice (Fig. 6a, b). Consistently, we found that the colony-forming capacity of *Casp8*^*−/−*^*Ripk3*^*−/−*^ BM cells was also comparable to that observed in both *WT* and *Ripk3*^*−/−*^ mice (Fig. 6c). To study whether *Ripk3* deletion can restore the self-renewal capacity of *Casp8*^*−/−*^ HSCs, we compared the CHRC of *WT, Casp8*^*−/−*^*, Ripk3*^*−/−*^ and *Casp8*^*−/−*^*Ripk3*^*−/−*^ HSCs using serial transplantation assays. We found that, consistent with our previous reports, *Rikp3*^*−/−*^ HSCs display improved CHRC compared to *WT* HSCs during serial transplantation [46]; however, the CHRC of *Casp8*^*−/−*^*Ripk3*^*−/−*^ HSCs is reduced compared to *WT* HSCs but is significantly improved compared to that of *Casp8*^*−/−*^ HSCs (Fig. 6d). Analyzing donor HSCs in recipient mice receiving a 3^rd^ transplantation, we found that significantly fewer donor HSCs were maintained in the *Casp8*^*−/−*^*Ripk3*^*−/−*^ transplantation compared to *WT* and *Ripk3*^*−/−*^ transplantations (Fig. 6e). It was known that inflammatory cytokines impair HSC self-renewal and function. To study whether impaired HSC function in *Casp8*^*−/−*^*Ripk3*^*−/−*^ mice is due to elevation of inflammatory cytokines, we compared the levels of BM inflammatory cytokines among *WT*, *Casp8*^*−/−*^*, Ripk3*^*−/−*^ and *Casp8*^*−/−*^*Ripk3*^*−/−*^mice. We found an increased IFNβ and reduced IL1β levels in BM of *Casp8*^*−/−*^*Ripk3*^*−/−*^ mice compared to other genotypes of mice (Figs. 6f and S6a), while the levels of all other cytokines were comparable among all genotypes of mice (Fig. S6b). A recent study suggested that *Casp8*^*−/−*^ HCs produce significantly more Type-I IFN when Ripk3 is deleted due to Ripk1-mediated Tbk1 activation [48]. Type-I IFN impairs self-renewal of HSCs via the activation of Stat1 signaling and increased mitochondrial ROS [49, 50]. We speculated that the impaired CHRC of *Casp8*^*−/−*^*Ripk3*^*−/−*^ HSCs might be due to the increased production of Type-I IFN. To test such a hypothesis, we examined the activities of Tbk1 and Stat1, mitochondrial Ros (mitoROS) levels as well as the expression of Type-I IFN-stimulated genes (ISG), *Oas2, Irf7* and *Ifnb* in HSCs. We found that Tbk1 and Stat1 activities are elevated in *Casp8*^*−/−*^*Ripk3*^*−/−*^ HSPCs compared to *WT, Casp8*^*−/−*^ and *Ripk3*^*−/−*^ HSPCs which are associated with increased mitoROS levels (Fig. 6g). We also demonstrated an increased expression of *Oas2, Irf7* and *Ifnb* in *Casp8*^*−/−*^*Ripk3*^*−/−*^ HSPCs compared to *WT* and *Ripk3*^*−/−*^ HPSCs (Fig. 6h). Importantly, Ripk1 or Tbk1 inhibition could suppress Stat1 activity, mitoROS levels and the expression *Oas2, Irf7* and *Ifnb* genes in *Casp8*^*−/−*^*Ripk3*^*−/−*^ HSPCs (Fig. 6i, j). However, *Mlkl* inhibition failed to do so in *Casp8*^*−/−*^*Ripk3*^*−/−*^ HSPCs (Fig. S7**)**. This suggests that the elevated Tbk1-Ifnb signaling and mitochondrial ROS levels in *Casp8*^*−/−*^*Ripk3*^*−/−*^ HSPCs are due to the activation of Ripk1 kinase and are independent of Mlkl.

### Mice transplanted with Casp8^−/−^ BM developed MDS-like disease within 4 months

**Fig. 7:**
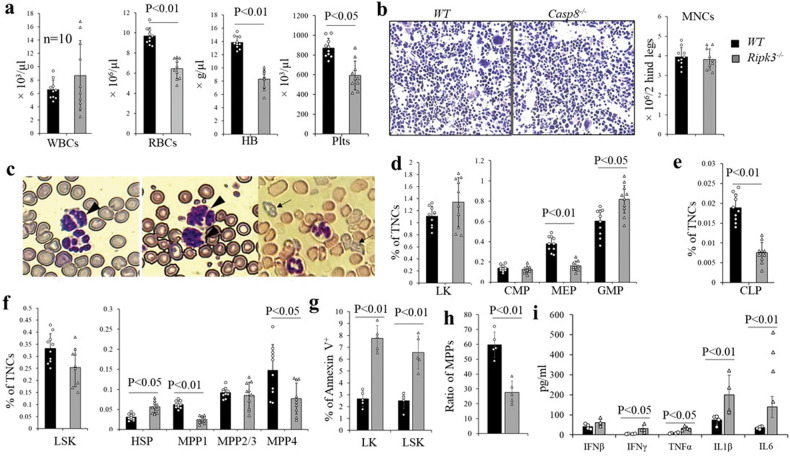
Mice transplanted with Casp8^−/−^ BM cells developed MDS like-diseases. Lethally-irradiated mice were transplanted with BM cells from either *WT* mice or *Casp8*^*−/−*^ mice, with each mouse receiving 2×10^6^ BM cells. All mice were terminated for analysis 4 months after transplantation. WBCs, RBCs, HB and platelet numbers in PB were determined by a Hemavet 950FS (**a**), BM cellularity was compared by histology analysis of BM sections and MNC counts (**b**); dysplasia of blood cells was indicated by Wright’s Giemsa staining of PB smears. Hypersegmented neutrophils (arrowhead) and polychromatic erythrocytes (arrow) were present. (**c**); percentages of LK population (including CMP, GMP, and MEP) (**d**), CLP (**e**) and LSK HSPCs (including HSCs, MPP1, MPP2/3 and MPP4) (**f**) in BM were examined by flow cytometry. Cell death in LK and LSK HSPCs was examined by flow cytometry for Annexin-V staining (**g**). HSCs (LSK-CD150^+^CD135^-^CD48^-^) were purified from BM by FACS and cultured in Stemspan serum-free medium supplemented with SCF, TPO and Flt3L. Percentages of MPPs were analyzed after 48 hours of culturing (**h**). Levels of inflammatory cytokines including IFNβ, IFNγ, TNFα, IL1β and IL6 were measured using LEGENDplex™ assay (**i**).

We found that some of the *Casp8*^*−/−*^ mice developed MDS-like disease 4 months after the induction of *Casp8* deletion as shown by anemia and thrombocytopenia with morphologic dysplasia of blood cells. Our *Casp8*^*−/−*^ mice are whole body knockouts. Therefore, to study the role of Casp8 specifically in hematopoiesis, we transplanted *Casp8*^*−/−*^ BM cells into lethally-irradiated recipient mice. Four months after transplantation, all recipient mice were terminated in order to perform hematopoietic analyses. We found that all 10 mice which had received *WT* BM cells were healthy with normal blood cell counts. However, 10/10 mice which had received *Casp8*^*−/−*^ BM cells developed a certain degree of anemia, 8/10 exhibited reduced platelet numbers and 4/10 displayed splenomegaly with increased WBC counts (Fig. 7a). BM cellularity in mice which had received *Casp8*^*−/−*^ BM cells was comparable to that in mice which had received *WT* BM cells (Fig. 7b). Hypersegmented neutrophils and polychromatic erythrocytes were observed in PB and BM of mice in the *Casp8*^*−/−*^ group suggesting dysplastic myeloid cell and red blood cell morphologies (Fig. 7c). The elevated granulocyte and monocyte counts but reduced lymphocyte counts in PB suggested myeloid-biased hematopoiesis (Fig. S8a). Detailed analysis of HSPCs in BM demonstrated a myeloid-biased and accelerated aging-related hematopoietic phenotype of mice which had received *Casp8*^*−/−*^ BM cells, as demonstrated by increased percentages of phenotypic HSC and GMP populations but reduced MPP1, MPP4, MEP and CLP populations (Fig. 7d-f). Increased death of LK and LSK HSPCs in *Casp8*^*−/−*^ BM cells suggested an ineffective hematopoiesis that is commonly observed in MDS BM (Fig. 7g). In addition, MPP differentiation of HSCs isolated from *Casp8*^*−/−*^ BM was significantly reduced compared to HSCs isolated from *WT* BM, suggesting an age associated HSC phenotype (Fig. 7h). The elevated Tnfα, IL1β, IL6 and Ifnγ in PB suggested an inflammatory reaction-associated pathogenesis (Fig. 7i). All these observations suggested an MDS-like disease in mice that had received *Casp8*^*−/−*^ BM cells. Such hematopoietic alterations were not due to the development of autoimmune lymphoproliferative syndrome (ALPS)-like disease because none of the mice displayed autoimmune-related features at the time of analysis, as shown by negativity for autoimmune antibodies (anti-nuclear antibody and anti-dsDNA) (Fig. S8b) and normal levels of immunoglobin (Fig. S8c), as well as negativity for CD4^−^CD8^−^CD3^+^B220^+^ double-negative T-cells (Fig. S8d).

## Discussion

Infections, which are largely caused by bacteria or fungi, are major contributors to morbidity and mortality in MDS patients. Such infections exacerbate these patients’ clinical symptoms by further impairing the processes involved in hematopoiesis [51]. Inflammatory cytokines such as TNFα, IFNγ and IL1β, as well as other death receptor agonists including FasL and S100A9, are elevated in MDS. Increased incidence of all three types of PCD, apoptosis, necroptosis and pyroptosis of HSPCs, are commonly detected in BM tissues of MDS patients [7–9]. Casp8, the master regulator of these three types of PCD, is down-regulated in BM HCs of MDS patients, which is associated with significantly increased concentrations of Ripk1 protein and Mlkl phosphorylation [4]. Mutations of the splicing factor SRSF2 are commonly detected in MDS, which lead to mis-splicing of *Casp8*, resulting in truncated forms of catalytically-dead Casp8 enzymes [10]. All these clinical observations suggest that Casp8 might be involved in the pathogenesis of MDS. As is the case with MDS patients, we found that *Casp8*^*−/−*^ mice are vulnerable to infections. The phenotype of our *Casp8*^*−/−*^ mice resembles most of the clinical characteristics of MDS patients, including cytopenia and myelodysplasia at 4 months after induction of *Casp8* deletion. By carefully comparing hematopoiesis among polyI:C and TAM-induced *Casp8*^*−/−*^ and *Casp8*^*−/−*^*Ripk3*^*−/−*^ mice, we uncovered a critical role for Casp8 in the regulation of HSPC survival and HSC self-renewal. Our study suggests that Casp8 is dispensable for homeostatic hematopoiesis in young adult mice but is essential in preventing Ripk3-mediated necroptosis during bacterial infections or inflammations. Casp8 also plays a critical role in regulating HSC function and self-renewal by restricting Ripk1-Tbk1-mediated Ifnβ production.

The role of Casp8 regulation of death-receptor signal-induced PCD has been well-documented. *Casp8*^*−/−*^ mice are embryonically lethal due to the activation of Ripk3/Mlkl-mediated necroptosis in vascular endothelial cells [11, 52]. The significant reduction of HSPCs in the aorta–gonad–mesonephros (AGM) region and fetal livers of *Casp8*^*−/−*^ embryos is most likely secondary to the vascular endothelial defects. The developmental defects observed in *Casp8*^*−/−*^ mice can be completely rescued by deletion of *Ripk3*, suggesting a critical role for Casp8 in restricting Ripk3-mediated necroptosis during development. *Casp8*^*−/−*^*Ripk3*^*−/−*^ mice develop ALPS within 4-5 months of age, resembling the phenotype of human ALPS, implicating a critical role for Casp8 in the restriction of autoimmune reactions [53, 54].

Studies of cell type-specific knockout mice suggested that Casp8 plays an essential role in regulating proliferation, mitosis/genetic stability, differentiation and cytokine production in different types of hematopoietic and immune cells. For example, in T cell-specific *Casp8*^*−/−*^ mice, peripheral T cells are significantly reduced due to defects in proliferation stimulated by cytokines, T cell receptor mitogens, or antigen-induced activation and immune responses [55, 56]. Myeloid-specific *Casp8*^*−/−*^ mice developed a mild systemic inflammation due to the increased numbers of Ly6C^high^ and Ly6C^low^ splenic CD11b^+^F4/80^+^ macrophages [57]. *Casp8*^*−/−*^ macrophages are hyperresponsive to TLR activation and exhibit aberrant M1 macrophage polarization [57]. *Casp8*^*−/−*^ natural killer cells have defects in accumulating in response to viral infection [58]. Interestingly, all these defects can be prevented by *Ripk3* deletion, suggesting a critical role for Casp8 signaling in restricting Ripk3-Mlkl-mediated necroptosis in these cell types [53, 57, 59, 60]. However, the defects in *Casp8*^*−/−*^ B cells and dendritic cells (DCs) cannot be prevented by *Ripk3* deletion. *Casp8*^*−/−*^ B cells display defects in TLR antagonist-induced proliferation and T-independent Type I Ab responses [61–63]. *Casp8*^*−/−*^ B cells also exhibit abnormal mitosis and impaired cytokinesis, which facilitate cellular transformation [64]. Approximately 50% of B cell-specific *Casp8*^*−/−*^ mice developed lymphomas with elevated chromosomal instability within 60 weeks [42]. *Casp8*^−/−^ DCs display a heightened costimulatory capacity and an elevated response to TLR signaling. DC-specific *Casp8*^−/−^ mice develop a systemic autoimmune disease which can be abrogated by Ripk1 inhibition and *Myd88* deletion but is exacerbated by *Irf3* deletion [65].

We found that the inflammation/infection-induced HSPC necroptosis and BM failure in *Casp8*^−/−^ mice can be completely prevented by *Ripk3* deletion; however, the HSC self-renewal defects of *Casp8*^−/−^ HSCs is mediated by Ripk1-Tbk1-Ifnβ signaling independent of Ripk3. In addition, the myelodysplastic phenotype observed in *Casp8*^−/−^ mice is also independent of Ripk3 because it can also be observed in some *Casp8*^−/−^*Ripk3*^−/−^ mice. We believe that such a myelodysplastic phenotype might be associated with the role of Casp8 in the regulation of mitosis, chromosomal stability and DNA damage repair [40, 42]. Future study needs to determine whether there are increased genetic mutations and chromosomal abnormalities in *Casp8*^−/−^ HSPCs. In addition, *Casp8* deletion or silencing has been reported in many types of solid cancers [66–72], and loss of Casp8 has been shown to facilitate cellular transformation in vitro [64, 73]. In hematopoietic malignancies, mutations of asp8 (within the P10 subunit) were reported in 58.21% (85/146) of AML cases, which are correlated with resistance to chemotherapy and poor prognosis for patients [74]. Thus, long-term observation is required to determine whether AML transformation occurs in *Casp8*^−/−^ mice.

The roles of Casp2, Casp3 and Casp9 in hematopoietic regulation have been studied. *Casp2*^*−/−*^ mice develop normally but show an ageing-associated phenotype with increasing oxidative stress, enhanced aneuploidy and DNA damage in their BM cells [75, 76]. Casp3 limits the sensitivity of HSPCs to Ras-Raf-Mer-Erk signaling induced by cytokines such as TPO, SCF and IL3 [77]. Mouse *Casp3*^*−/−*^ HSCs exhibit phenotypes including premature exit from quiescence, over-proliferation and retarded differentiation due to reduced Erk signaling [77]. Casp3 also regulates B cell proliferation and differentiation, as well as the maturation of erythroid blasts and megakaryocytes [78, 79]. *Casp9*^*−/−*^ HSCs display impaired self-renewal and CHRC due to the increased production of Ifnβ [80], which recapitulates the phenotype of our *Casp8*^*−/−*^*Ripk3*^*−/−*^ HSCs despite the different mechanisms involved in the activation of Tbk1-Irf3 signaling. In *Casp9*^*−/−*^ HSPCs, mtDNA released from mitochondria stimulates cGas/Sting-Tbk1 signaling and Ifnβ production, while in *Casp8*^*−/−*^*Ripk3*^*−/−*^ HSPCs, elevated Ripk1/Tbk1 interaction stimulates Tbk1 activation and Ifnβ production. In both situations, Ifnβ impairs HSC self-renewal by triggering mitoROS generation. Elevated Ifnβ levels in serum and *ISG* gene expression in BM cells were reported in MDS patients [81]. Future study must determine whether Tbk1 is also activated in MDS HSPCs and contributes to impaired HSC function in MDS patients.

## Materials and methods

### Generation of inducible Casp8 knockout mice and Casp8/Ripk3 compound-mutant mice

*Casp8*^*fx/fx*^ (*B6.129-Casp8tm1Hed/J*, Strain #:027002) [63], *Mx1Cre* (*B6.Cg-Tg(Mx1-Cre)1Cgn/J*, Strain #:003556) and *RosaCreERT* (*B6.129-Gt(ROSA)26Sortm1(Cre/ERT2)Tyj/J*, Strain #:008463) mice were purchased from JAX Laboratories. All mice were maintained in specific pathogen-free (SPF) facilities as indicated according to the standards set forth in the National Institutes of Health Guidelines for the Care and Use of Animals in the animal facility at Loyola University Medical Center and at Lanzhou University Second Hospital in a CD57B6 background. All experiments performed on animals were approved in advance by the Loyola University Institutional Animal Care and Use Committee (protocol No. 108832) or the Lanzhou University Institutional Animal Care and Use Committee. By crossing *Mx1Cre* mice or *RosaCreERT* mice with *Casp8*^*fx/fx*^ mice and then back-crossing, we generated interferon-inducible *Casp8* knockout mice (*Mx1Cre*^*+*^*Casp8*^*fx/fx*^) and tamoxifen (TAM)-inducible *Casp8* knockout mice (*RosaCreERT*^*+*^*Casp8*^*fx/fx*^), respectively, and also corresponding control mice (*Mx1Cre*^*+*^*Casp8*^*fx/+*^ and *Casp8*^*fx/fx*^). To induce *Casp8* deletion, 6–8 wks. after birth, all littermates in *Mx1Cre* groups were injected with polyI:C, 5 μg/g body weight, every other day for a total of three injections; mice in the *RosaCreERT*^*+*^ groups were given 100 mg/kg/day of TAM by intraperitoneal injection for 5 consecutive days. By back-crossing *Rosa26CreERT*^*+*^*Casp8*^*fx/fx*^ or *Mx1Cre*^*+*^*Casp8*^*fx/+*^ mice with *Ripk3*^*−/−*^ mice, we generated *RosaCreERT*^*+*^*Casp8*^*fx/fx*^*Ripk3*^*−/−*^ and *Mx1Cre*^*+*^*Casp8*^*fx/fx*^*Ripk3*^*−/−*^ mice, respectively, as well as corresponding controls. PolyI:C or TAM was injected to induce *Casp8* deletion as described above. Mice genotyping was accomplished by PCR analysis of tail snip DNA using primers listed in supplementary Table 1. The efficiency of *Casp8* deletion in *Casp8*^−/−^ mice was determined by PCR assays using the primers listed in supplementary Table 1. To assess the responses of mice to polyI:C or LPS treatment, some of them were treated with 1 μg/g body weight of polyI:C or 0.2 μg/g body weight of LPS every other day for 16 days, as indicated.

### Mouse hematopoietic phenotype analysis

Mice were humanely terminated at the indicated time points to collect peripheral blood, spleens, thymuses, and BM. PB was analyzed for WBC counts, platelet counts, RBC counts, and Hb concentration using a Hemavet 950FS (Drew Scientific Inc.). After lysis of RBCs, nucleated cells from PB, spleens, thymuses, and BM were counted and further stained with cell surface markers for phenotypic analysis by flow cytometry as described previously [46]. All the fluorescent antibodies used in flow cytometric analyses were purchased from Biolegend or eBioscience and are listed in supplementary Table 2. Stained cells were subjected to multi-color analysis using a BD LSR-Fortessa^TM^ flow cytometer. Data were analyzed using Flowjo software. All small molecular chemicals and cytokines used in this study are listed in supplementary Table 3.

### Statistical analyses

The statistical analyses of all the data presented in this manuscript were performed using GraphPad Prism-9 software. Two-way ANOVA (multiple groups) and Student’s t tests (two groups) were performed to determine the statistical significance of differences among and between experimental groups. Statistical data are expressed as means ± SD. Mantel–Cox log-rank test was performed for Kaplan–Meier survival comparison between experimental groups. *p* < 0.05 was considered to be statistically significant.

Please find other detailed information of materials and methods in the supplementary files.

### Supplementary information

Supplementary data

Original Western blot data

Orignal data file

## Data Availability

All the original data and experimental materials related to this paper are available upon request.
