# Supplementary material for: Caspase 8 deletion causes infection/inflammation-induced bone marrow failure and MDS-like disease in mice
Source: Cell Death Dis. 2024 Apr 18;15(4):278. doi: 10.1038/s41419-024-06660-3 (PMC11026525; doi:10.1038/s41419-024-06660-3)

**Caspase 8 deletion causes infection/inflammation-induced bone marrow failure**

**and MDS-like disease in mice**

Shanhui Liu^1,2,3,#^, Kanak Joshi^1,2,#^, Lei Zhang^1,2,4^, Wenyan Li^3^, Ryan Mack^1,2^, Austin Runde^1,2^, Patrick A. Hagen^1,5^, Kevin Barton^1,5^, Peter Breslin^1,2,6^, Hong-Long Ji^7^, Ameet R. Kini^8^, Zhiping Wang^3*^, Jiwang Zhang^1,2,8*^

**Materials and methods**

### *Cell lysate collection and Western blot analysis.* After designated treatments, cells were collected from culture by centrifugation (2000 rpm × 5 mins) to remove the culture medium and were then re-suspended into 1× SDS lysis buffer. Suspensions were briefly sonicated for 5 pulses and subsequently centrifuged at 14,000 rpm × 15 min. at 4 °C., followed by supernatant purification. Lysates were either used immediately or stored at -80°C. until use. Quantities of 20-30µg of protein per sample were loaded onto freshly-made 1.5-mm gels (8-14% acrylamide) alongside 2-3µL of protein molecular weight standard, and electrophoresis was performed at 60–65 V. for 10-15 mins., then at 120-140 V. for 80-90 mins. Size-fractionated protein samples were immediately transferred onto a nitrocellulose membrane using the Thermo Fisher TurboTransfer System in accordance with equipment use protocols, followed by a brief ddH_2_O wash of each membrane. Membranes were blocked using 3% BSA in 1× TBST for at least 60 mins. and incubated overnight at 4 °C. on a shaker platform with the primary antibodies specified (1:500-1:1000 dilution in fresh blocking agent). The antibodies used are listed in Table 2. Membranes were then washed with 1× TBST five times for 5-10 mins. each and then incubated with secondary antibody for 30-60 mins. (1:1250 dilution in fresh blocking agent). Membranes were washed again 3-5 times for 10-20 mins. in TBST and subsequently imaged using a BioRad Imager at various exposure times. For re-blotting purposes, membranes were warmed in TBST for 10 mins. and stripped (if applicable) with 1× Thermo Fisher Stripping Buffer for 20-30 mins., followed by 2-3 TBST washes for 10 mins. each and re-blocked for at least 30 mins. New primary antibodies were used at the same dilution conditions specified above for at least 60 mins. All subsequent steps for re-blotting were identical to those described above.

***Competitive BM serial transplantation***. Equal numbers of BM cells from *WT, Casp8^-/-^, Ripk3^-/-^, or Casp8^-/-^Ripk3^−-/-^* (CD45.2^+^) were mixed with equal numbers of competitor BM cells from *Ptprc* mice (CD45.1^+^), respectively, to reach a ratio of 50:50. The mixed BM cells were transplanted into lethally irradiated *Ptprc* (CD45.1^+^) recipient mice. Each recipient mouse received 2×10^6^ mixed BM cells. Six mice were transplanted for each genotype of BM cells. Ten wks. after transplantation, all recipient mice were terminated and the contribution of donor HSCPs to hematopoiesis in the recipients was assessed by analyzing the CD45.2^+^ cell percentages in the BM and PB. Subsequently, a mixture of BM cells from all 6 mice from each group were transplanted into a new group of six lethally irradiated *Ptprc* mice, with each mouse receiving 2×10^6^ mixed BM cells. Hematopoietic engraftment in 2^nd^ recipient mice was analyzed 10 wks. later and a mixture of BM cells from each group of mice was further transplanted into 3^rd^ recipient mice as described above.

***BM transplantation for MDS development***. BM cells from *WT* or *Casp8^-/-^* (CD45.2^+^) were transplanted into lethally irradiated *Ptprc* (CD45.1^+^) recipient mice. Each recipient mouse received 2×10^6^ mixed BM cells. Ten mice were transplanted for each genotype of BM cells. All recipient mice were monitored and terminated 4 months later for hematopoietic analysis to determine disease development.

### *RNA extraction and reverse transcription–polymerase chain reaction (RT-PCR)*.  RNA was extracted from indicted cells using Tri Reagent following the instructions provided by the vendor (Invitrogen). RNA extracts were quantified via NanoDrop 2000, and 2µg of RNA from each sample was treated with DNAse I (1U/µL) in 1× DNAse I buffer at 37°C. for 30 mins., followed by 2.5mM EDTA treatment at 65°C. for 10 mins. to remove DNA. Reverse transcription (RT) was then conducted to generate cDNAs using SuperScript^®^ III Reverse Transcriptase (Life Technologies) in accordance with the supplied protocol. The expression of the genes of interest in each cDNA sample was examined by TaqMan™ qRT-PCR Assay. Information on the primers and probes used for each gene is listed in Supplementary Table 1. The relative levels of gene expression are presented as 2^-ΔΔCT^, which was calculated using the equation: ΔΔCT = (Ct_GOI_- Ct_refer_)-average of ΔCT of control samples.

### *Analysis of inflammatory cytokines and autoantibody*. BM fluids were collected from the four long bones (two femurs and two tibiae) of each mouse by flushing with the same 200μl of HBSS using a 1ml insulin syringe with a 27G. needle and spun at 500 × g for 5 mins. to remove BM cells. Supernatants were further clarified by centrifugation at 12,000 × g. for 10 mins. PB was collected from mice by cardiac stick and allowed to clot, and serum was separated out by centrifugation. Samples were subsequently stored at -80°C until use. Serum cytokine concentration was measured using LEGENDplex™ Mouse Inflammation Panel (13-plex) with V-bottom plates (Biolegend) according to the manufacturer’s instructions. The concentrations of cytokines were analyzed using LEGENDplex Data Analysis Software. Serum autoantibodies for dsDNA (IgM and IgG) and anti-nuclear antibodies (ANAs, total Ig) with ELISA, as described by the manufacturer (Supplementary Table 2). Serum immunoglobulin levels were analyzed using BD™ Cytometric Bead Array (CBA) Mouse Immunoglobulin Isotyping Kit (Supplementary Table 2).

***Colony-forming unit assay (CFU) and treatments***. Murine BM MNCs were seeded at 20,000 cells/mL into MethoCult GF M3434 medium for CFU assay following the instructions provided by the vendor (StemCell Technologies) with or without treatments, as indicated. For treatments of the cells in CFU assay, 100-300ng/ml LPS, 10-50μg/ml polyI:C, 20-50ng/ml TNFα, 20-50ng/ml IFNα, 20-50ng/ml IFNγ were added to the medium and mixed well before the cells were added. Information on the chemicals used can be found in **Supplementary Table 3**.

***Annexin-V and 7-AAD staining to analyze for apoptosis***. BM MNCs were collected from mice with indicated genotypes. c-Kit^+^ HSPCs and c-Kit^-^ HCs were isolated using MojoSort™ Mouse CD117 (c-Kit) Selection Kit following the vendor’s instructions. Cells then were treated with the indicated chemicals and stained with allophycocyanin-conjugated Annexin-V followed by 7-amino-actinomycin D (7-AAD) staining in binding buffer following the manufacturer’s instructions (BD Biosciences). The death of infected cells was examined by analyzing the percentages of Annexin-V^+^ and Annexin-V^+^/7-AAD^+^ cells by flow cytometry.

***Intracellular protein analysis***. c-Kit^+^ HSPCs and c-Kit^-^ HCs BM cells from mice with the indicated genotypes were collected into lysis/fixation buffer (BD Biosciences) to simultaneously fix the nucleated cells and lyse the RBCs. After two washes with cold PBS/2% FBS, BM cells were stained with cell surface markers as indicated for 30 mins. Cells were then permeabilized using a fixation/permeabilization kit (BD Biosciences) for 20 mins. at room temperature. Cells were next washed with wash buffer twice and stained with antibodies against intracellular proteins, followed by APC-conjugated secondary antibody staining. The levels of intracellular proteins were detected by flow cytometry comparing the APC intensity in different populations of BM cells. PE-conjugated rabbit anti-mouse cleaved Casp8 (Asp387), rabbit anti-mouse p-Ripk1 (Ser166), rabbit anti-mouse p-Ripk3 (Thr231/Ser232), and rabbit anti-mouse p-Mlkl (Ser345) antibodies were purchased from Cell Signaling Technology (**Supplementary Table 2**).

**Supplementary Data**

***Supplementary Table 1. List of PCR primers used in this study.***

| Genes | Primers | Products |
| --- | --- | --- |
| *Casp8 F* | GAG AAT ATA ATT CCC CCA AAT CCT C | Genotype |
| *Casp8 R1* | AGT CAC AGC AGG GCT CAC T | Wild type = 210 bp; fx = ~325 bp |
| *Casp8 R2* | GCCATCTACCACAGAGCACA | Deletion= 810bp |
| *Cre F* | GCG GTC TGG CAG TAA AAA CTA TC | Genotype |
| *Cre R* | GTG AAA CAG CAT TGC TGT CAC TT | ~100 bp |
| Internal positive control F | CTA GGC CAC AGA ATT GAA AGA TCT | Genotype |
| Internal positive control R | GTA GGT GGA AAT TCT AGC ATC C | 324 bp |
| *Ripk3 F* | CGCTTTAGAAGCCTTCAGGTTGAC | Genotype |
| *Ripk3 R1* | GCAGGCTCTGGTGACAAGATTCATGG | Wild-type=700bp, Mutant=450bp |
| *Ripk3 R2* | CCAGAGGCCACTTGTGTAGCG | Genotype |
| Casp8 | Mm01255716_m1 | Thermofisher Scientific |
| Oas2 | Mm00460961_m1 | Thermofisher Scientific |
| Irf7 | Mm00516793_g1 | Thermofisher Scientific |
| Ifnb1 | Mm00439552_s1 | Thermofisher Scientific |

***Supplementary Table 2. List of Antibodies used in this study.***

| **Antibodies** | **Catalog #** | **Venders** |
| --- | --- | --- |
| Caspase-8 (D35G2) Rabbit mAb | #4790 | Cell Signaling Technology |
| Caspase-3 Antibody | #9662 | Cell Signaling Technology |
| RIPK1 (D94C12) XP^®^ Rabbit mAb | #3493 | Cell Signaling Technology |
| Phospho-RIPK1 (Ser321) (E9K2A) Rabbit mAb | #38662 | Cell Signaling Technology |
| RIPK3 (D4G2A) Rabbit mAb | #95702 | Cell Signaling Technology |
| Phospho-RIP3 (Thr231/Ser232) (E7S1R) Rabbit mAb | #91702 | Cell Signaling Technology |
| MLKL (D6W1K) Rabbit mAb | #37705 | Cell Signaling Technology |
| Phospho-MLKL (Ser345) (D6E3G) Rabbit mAb | #37333 | Cell Signaling Technology |
| GAPDH (D16H11) XP^®^ Rabbit mAb | #5174 | Cell Signaling Technology |
| FLIP (D5J1E) Rabbit mAb | #56343 | Cell Signaling Technology |
| APC anti-mouse CD117 (c-Kit) Antibody | 105812 | BioLegend |
| PE anti-mouse CD117 (c-Kit) Antibody | 105808 | BioLegend |
| APC anti-mouse Ly-6G/Ly-6C (Gr-1) Antibody | 108412 | BioLegend |
| Brilliant Violet 421™ anti-mouse Ly-6G/Ly-6C (Gr-1) Antibody | 108434 | BioLegend |
| APC anti-mouse/human CD45R/B220 Antibody | 103212 | BioLegend |
| Pacific Blue™ anti-mouse/human CD45R/B220 Antibody | 103227 | BioLegend |
| FITC anti-mouse CD3 Antibody | 100204 | BioLegend |
| Pacific Blue™ anti-mouse CD3 Antibody | 100214 | BioLegend |
| PE anti-mouse TER-119/Erythroid Cells Antibody | 116208 | BioLegend |
| PE/Cyanine5 anti-mouse TER-119/Erythroid Cells Antibody | 116210 | BioLegend |
| Brilliant Violet 421™ anti-mouse TER-119/Erythroid Cells Antibody | 116234 | BioLegend |
| Brilliant Violet 785™ anti-mouse CD150 (SLAM) Antibody | 115937 | BioLegend |
| PE/Cyanine7 anti-mouse CD150 (SLAM) Antibody | 115914 | BioLegend |
| APC/Cyanine7 anti-mouse CD48 Antibody | 103432 | BioLegend |
| Brilliant Violet 785™ anti-mouse CD48 Antibody | 103449 | BioLegend |
| APC/Cyanine7 anti-mouse CD16/32 Antibody | 101328 | BioLegend |
| PerCP/Cyanine5.5 anti-mouse CD16/32 Antibody | 101324 | BioLegend |
| PE/Cyanine7 anti-mouse CD34 Antibody | 119326 | BioLegend |
| Brilliant Violet 650™ anti-mouse Ly-6A/E (Sca-1) Antibody | 108143 | BioLegend |
| PE anti-mouse Ly-6A/E (Sca-1) Antibody | 108108 | BioLegend |
| FITC anti-mouse CD41 Antibody | 133904 | BioLegend |
| Brilliant Violet 421™ anti-mouse CD41 Antibody | 133912 | BioLegend |
| Caspase-8 (active) FITC Staining Kit | ab65614 | Abcam |
| Cleaved Caspase-3 (Asp175) (D3E9) Rabbit mAb (Alexa Fluor® 647 Conjugate) | #9602 | Cell Signaling Technology |
| Cleaved Caspase-8 (Asp387) (D5B2) XP^®^ Rabbit mAb (PE Conjugate) | #14071 | Cell Signaling Technology |
| Phospho-RIPK3 (Thr231/Ser232) (E7S1R) Rabbit mAb | #91702 | Cell Signaling Technology |
| Phospho-RIPK1 (Ser166) Rabbit Antibody | #31122 | Cell Signaling Technology |
| Phospho-MLKL (Ser345) (D6E3G) Rabbit mAb | #37333 | Cell Signaling Technology |
| Goat anti-Rabbit IgG (H+L) Cross-Adsorbed Secondary Antibody, APC | A-10931 | ThermoFisher Scientific |
| MojoSort™ Mouse CD117 (c-Kit) Selection Kit | 480146 | BioLegend |
| LEGENDplex™ Mouse Inflammation Panel (13-plex) with V-bottom Plate | 740446 | BioLegend |
| LEGEND MAX™ Mouse IFN-β ELISA Kit | 439407 | Biolegend |
| Mouse Anti-dsDNA ELISA Kit | DEIA-S1021 | Creative Diagnostics |
| Mouse ANA (Anti-nuclear Antibody) ELISA Kit | DEIA-BJ2332 | 0.469-30ng/ml |
| BD™ Cytometric Bead Array (CBA) Mouse Immunoglobulin Isotyping Kit | 550026 | BD Biosciences |

***Supplementary Table 3. List of small molecular chemicals and cytokines used in this study.***

| **Chemicals** | **Catalog #** | **Venders** | **Doses** |
| --- | --- | --- | --- |
| Necrostatin 2 racemate (Nec-1s) | S8641 | Selleckchem | 10-30μM  2μg/g |
| GSK'872 | S8465 | Selleckchem | 3μM |
| GSK8612 (Tbki) | S8872 | Selleckchem | 5μg/g |
| Tamoxifen (TAM) | S1238  CAS 10540–29-1 | Selleckchem  SantaCruz Biotechnology | 100μg/g/day i.p. |
| Recombinant Mouse TNF-α (Animal-Free) | 718004 | Biolegend | 20-500ng/ml |
| Recombinant Mouse IFN-α (carrier-free) | 752806 | Biolegend | 20-100ng/ml |
| Recombinant Mouse IFN-γ (carrier-free) | 575306 | Biolegend | 20- 10 ng/mL |
| Polyinosinic-polycytidylic acid (polyI:C) | 27473201 | GE Healthcare | 1-5μg/g body weight  10-50μg/mL |
| Lipopolysaccharide (LPS) | L2880  Escherichia coli 0111:B4 | Sigma-Aldrich | 100-1000ng/mL  0.2μg/g |
| GW806742X | HY-112292 | MedChemExpress | 2-5μM *in vitro*  2mg/kg *in vivo* i.p. injection |

**Figure S1. *The levels of Casp1, Casp3, Casp7, Gsdm and Parp1 protein are comparable between*** ***c-Kit^+^ HSPCs and c-Kit^-^ HCs****.* Levels of Casp1, Casp3, and Casp7 as well as levels of GsdmD and Parp1 were compared between c-Kit^+^ HSPCs and c-Kit^-^ HCs from WT mice by Western blotting assay.

**
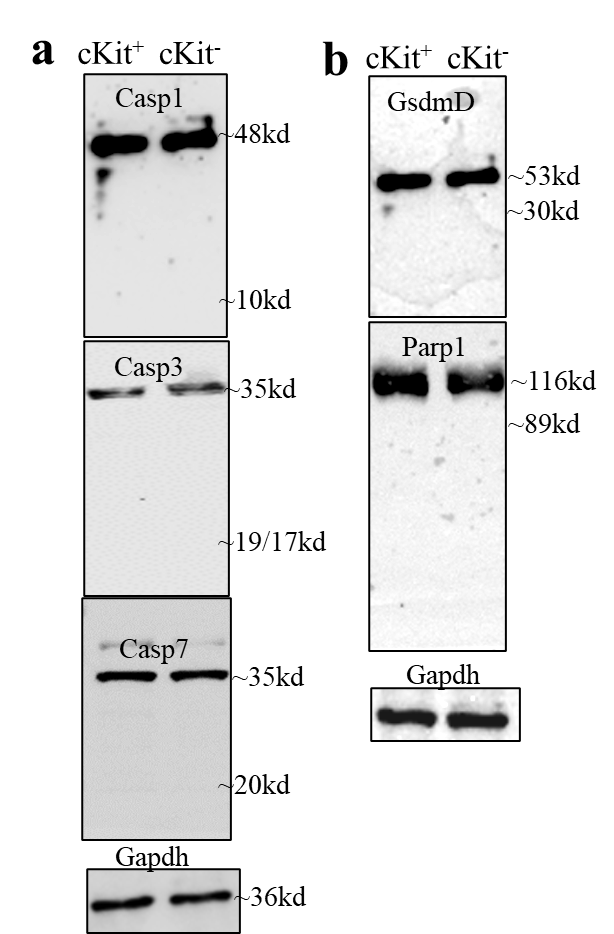
**

**Figure S2. *Mx1Cre+Casp8^fx/fx^* mice died within 12 days post 3 × polyI:C injections.** Eight weeks after birth, *Casp8^fx/fx^*, *Mx1Cre^+^Casp8^fx/+^* and *Mx1Cre^+^Casp8^fx/fx^* mice were treated with 5μg/g body weight of PolyI:C, every other day for a total of three injections to induce *Casp8* deletion. Survival of the mice were recorded by Kaplan–Meier estimator. Eight mice were studied in each group.


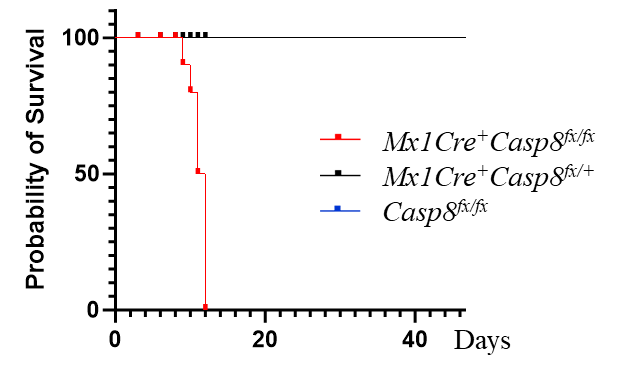


**Figure S3. Comparing Casp8 expression in BM HSPCs between *WT* and *Mx1Cre^+^Casp8^fx/+^* mice**. c-Kit^+^ HSPCs were isolated from *WT* and *Mx1Cre^+^Casp8^fx/+^* mice 10 days post polyI:C injections. Casp8 expression was examined by qRT-PCR (**a**) and Western blotting (**b**). Data in **a** is a summary of 3 mice in each group.

**
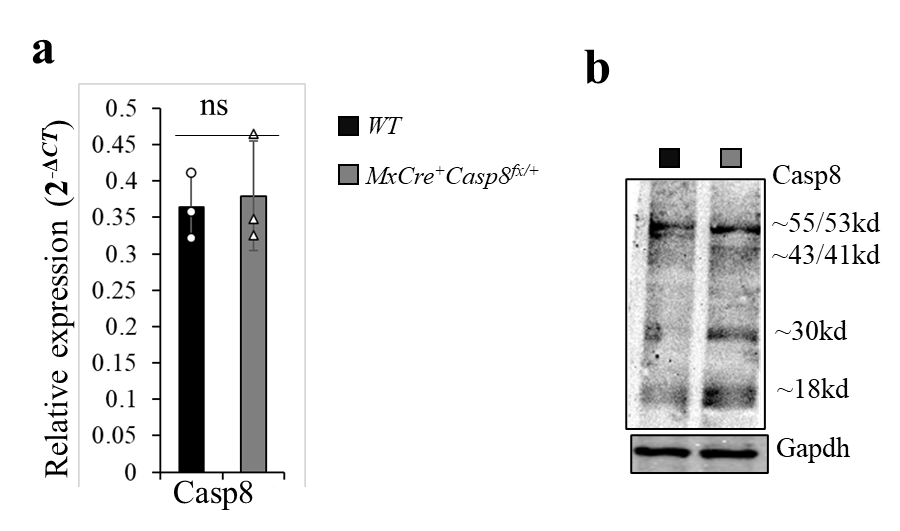
**

**Figure S4. Gating Strategy for flow cytometric analysis of HSPCs**. Hematopoietic stem cells (HSCs), multipotent progenitor 1 (MPP1) cells, multipotent progenitor 2/3 (MPP2/3) cells, multipotent progenitor 4 (MPP4) cells, common myeloid progenitors (CMP), megakaryocyte-erythroid progenitor s(MEP) and granulocyte-monocyte progenitors (GMP).

**
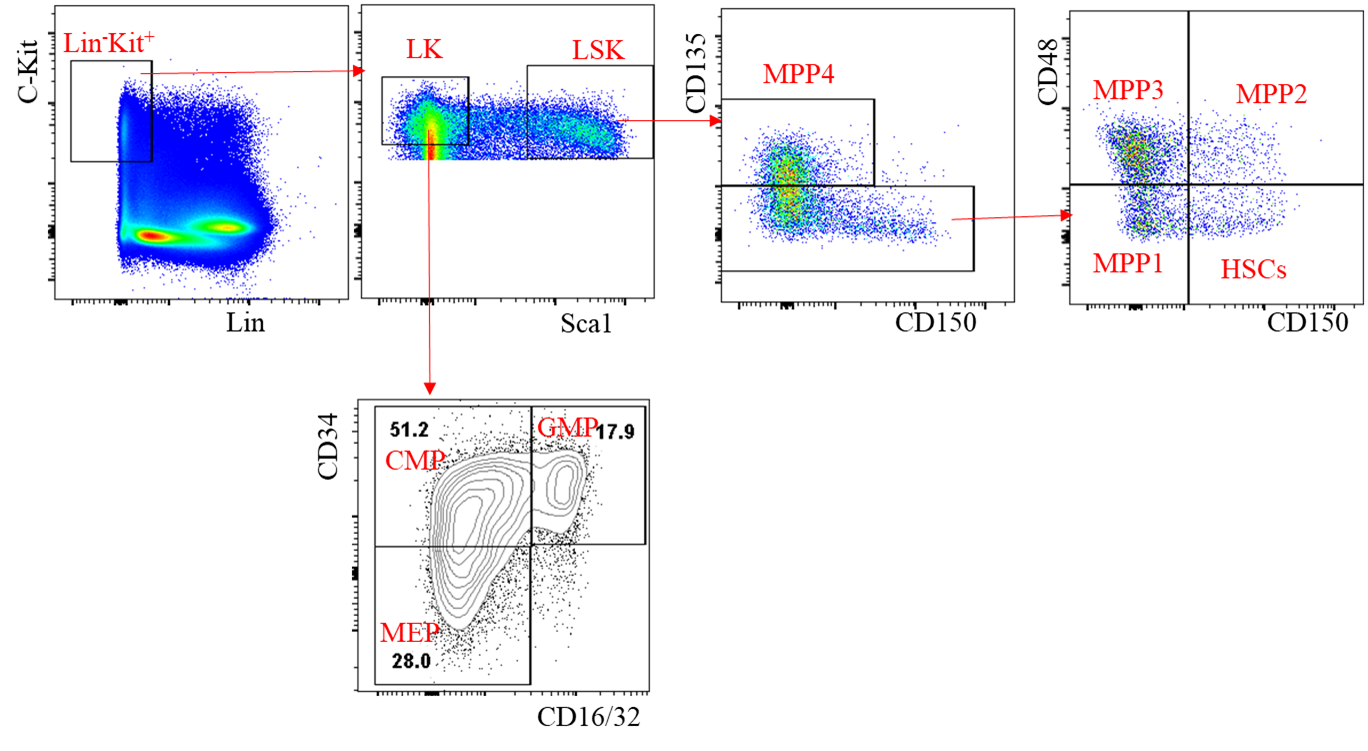
**

***Figure S5. PolyI:C/LPS and inflammatory cytokine-induced cell death in Casp8^-/-^ HSPCs can be prevented by Mlkl inhibition.*** cKit^+^ HSPCs were isolated from *WT* and *Casp8^-/-^* mice and cultured in HSPC culture medium. **a.** Cells were pretreated with 2μM Mlkl inhibitor GW806742X (GW) or vehicle for 1 hour and then treated with LPS (300ng/ml), polyI:C (50μg/ml), TNFα (50ng/ml), IFNγ (50ng/ml) or IFNα (50ng/ml) treatment. Cell death was examined by Annexin-V staining 24 hrs. after culturing (a). **b.** Cells were pretreated with 2μM GC or vehicle for 1 hour and then seeded into methylcellulose medium for colony-forming unit assay with or without LPS (300ng/ml), polyI:C (50μg/ml), TNFα (50ng/ml), IFNγ (50ng/ml) or IFNα (50ng/ml) treatment; CFU were counted on day 8 of culturing. Data represents one of the three independent experiments performed in triplicate. * and ** p<0.05 and <0.01, respectively compared to other groups. NS stands for no significance.


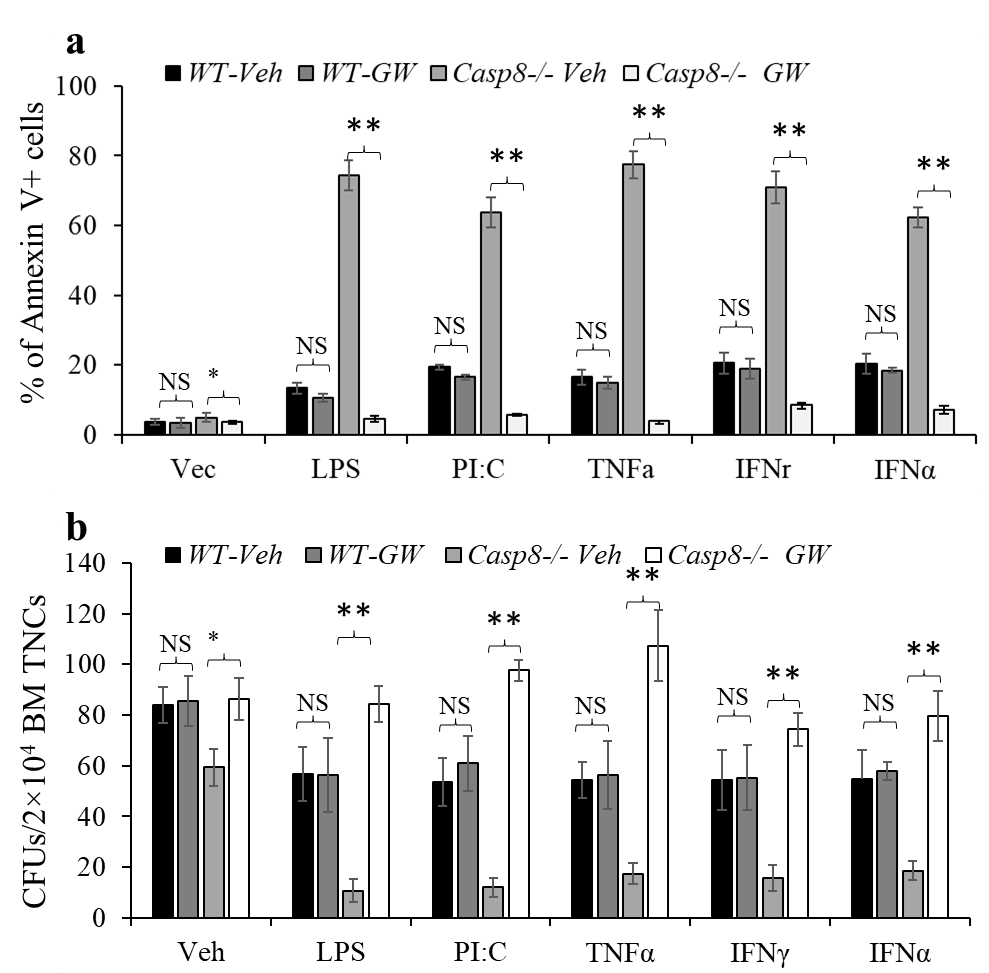


**Figure S6. The concentration of inflammatory cytokines in BM sera were compared among *WT, Ripk3^-/-^, Casp8^-/-^ and Casp8^-/-^Ripk3^-/-^*** (associated with Fig. 6)**.** BM sera were collected from the mice and the cytokine concentrations were measured using LEGENDplex™ Mouse Inflammation Panel (13-plex).


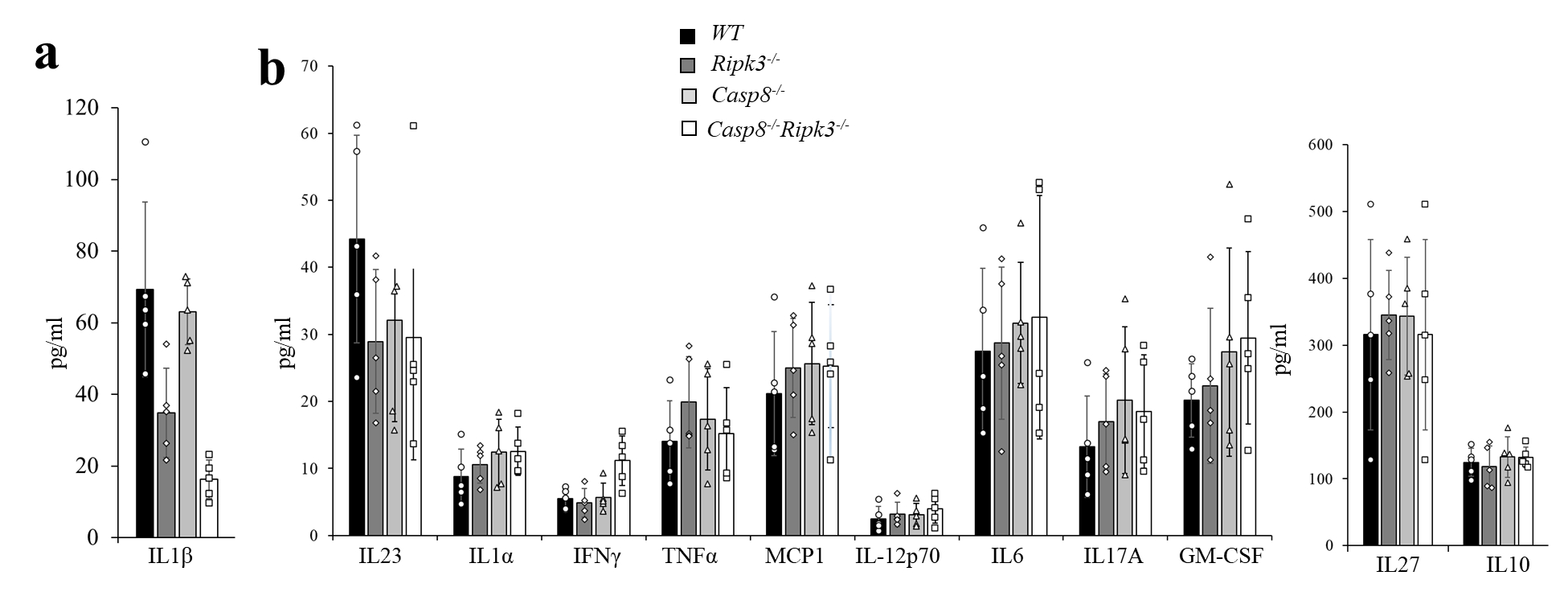


***Figure S7. Mlkl inhibition fails to prevent p-Tbk1-Ifn signaling in Casp8^-/-^ripk3^-/-^ HSCs.* a.** *Casp8^-/-^* mice were pre-treated with indicated concentrations of GW806742X (GW) or vehicle for 1 hour followed by polyI:C (1μg/g) treatment. BM mononuclear cells (MNCs) were collected 12 hrs. post-treatment and death of MNCs was examined by Annexin-V staining. ≥2mg/kg GW injection prevented polyI:C-induced death of *Casp8^-/-^* HSPCs *in vivo,* suggesting Mlkl inhibition. **b-c**. *Casp8^-/-^Ripk3^-/-^* mice were pre-treated with 2mg/kg GW or vehicle (Veh) for 2 days. cKit^+^ HSPCs were isolated from the mice 12 hrs. post-treatment; pTbk1, pStat1 and mito-ROS levels were examined by intracellular antibody or MitoSOX staining (**b**); the expression of ISG genes was examined by RT-PCR assay (**c**). Data in **a** represents an average value of 3 mice in each group. MFI ratio in **b** was counted from 3 independent experiments and normalized to WT controls. Data in **c** is a summary of 5 mice in each group. NS stands for no significance.

**
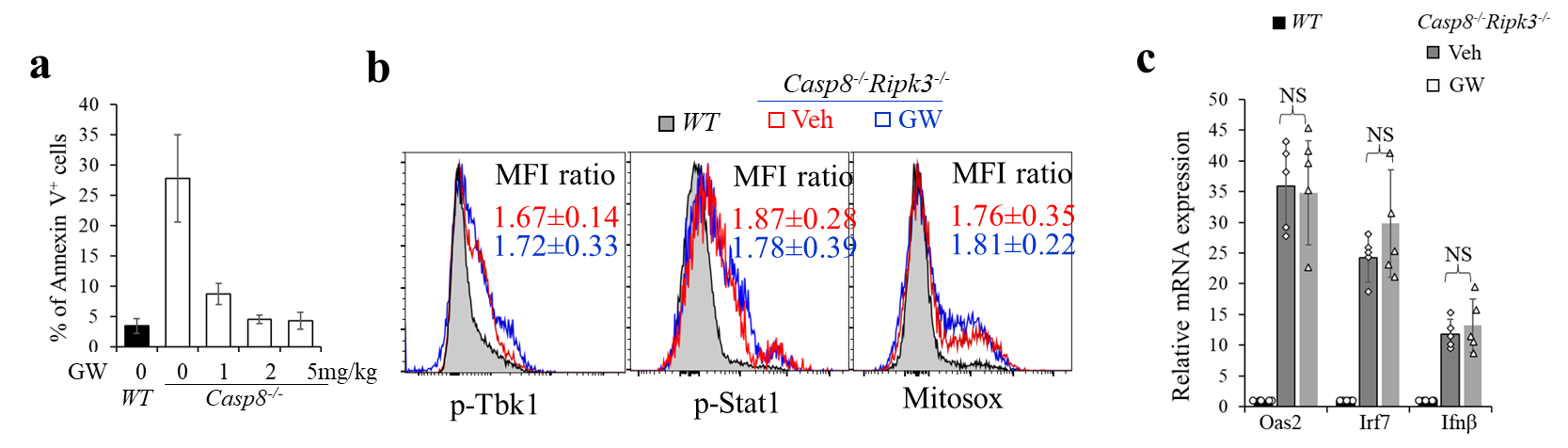
**

**Figure S8. *Mice transplanted with Casp8^-/-^ BM cells developed MDS-like disease four months after transplantation*** (associated with Fig. 7)***.* a.** Neutrophil, monocyte, and lymphocyte numbers in PB were determined by a Hemavet 950FS. **b**. Auto-antibodies ANA (anti-nuclear antibody) and anti-dsDNA (double-stranded DNA) were measured by ELISA. **c.** Immunoglobin concentrations in PB sera were examined by ELISA. Sera from *Casp8^-/-^Ripk3^-/-^* mice were used as controls. **d.** CD4^-^CD8^-^CD3^+^B220^+^ double-negative T-cells in PB were examined by flow cytometry and compared. PB from *Casp8^-/-^Ripk3^-/-^* mice was used as controls.


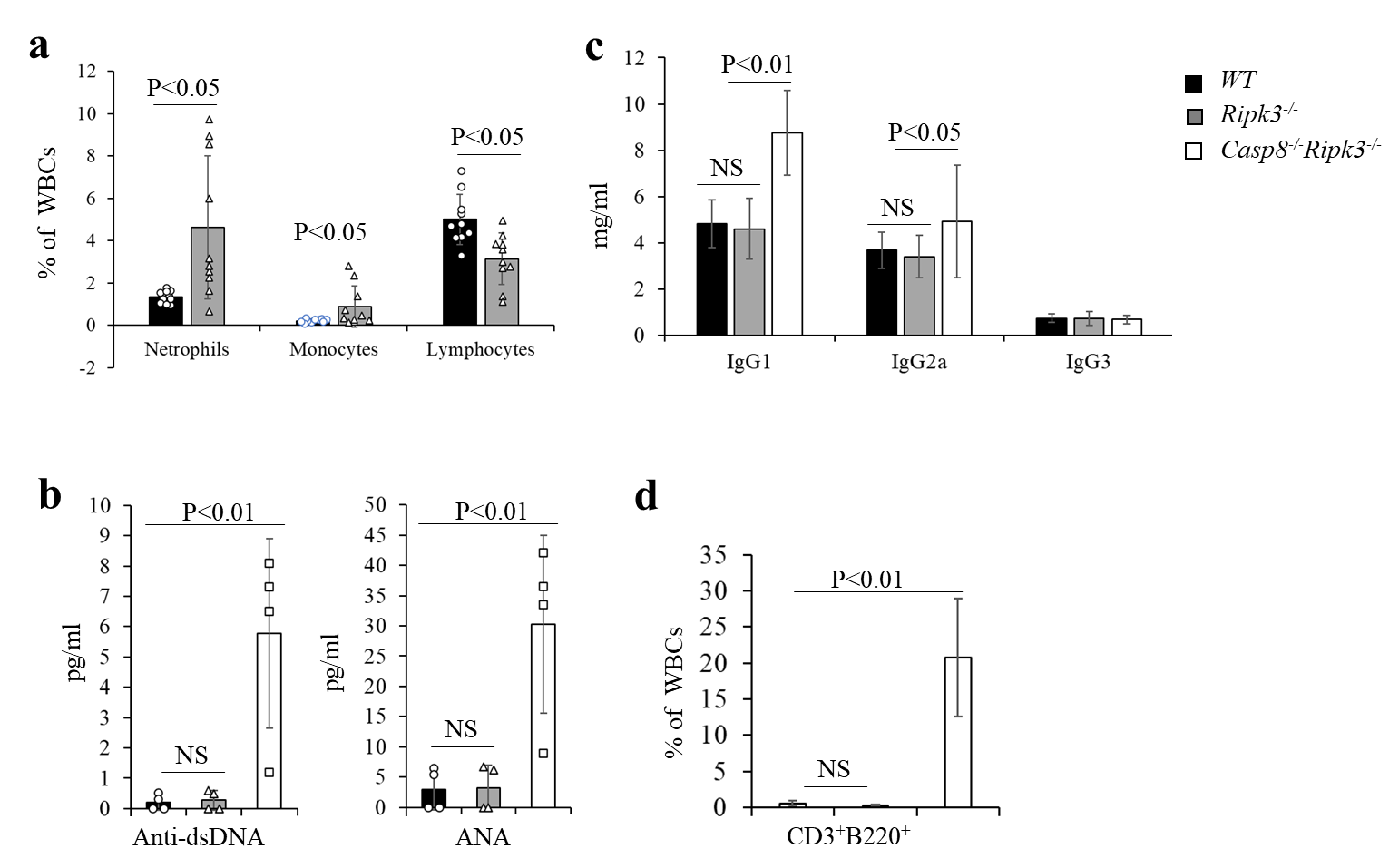

Supplement: Supplementary file 1 — Supplementary data [file 41419_2024_6660_MOESM1_ESM.docx]
