# Supplementary material for: Caspase 8 deletion causes infection/inflammation-induced bone marrow failure and MDS-like disease in mice
Source: Cell Death Dis. 2024 Apr 18;15(4):278. doi: 10.1038/s41419-024-06660-3 (PMC11026525; doi:10.1038/s41419-024-06660-3)

Original western blot data for figure1


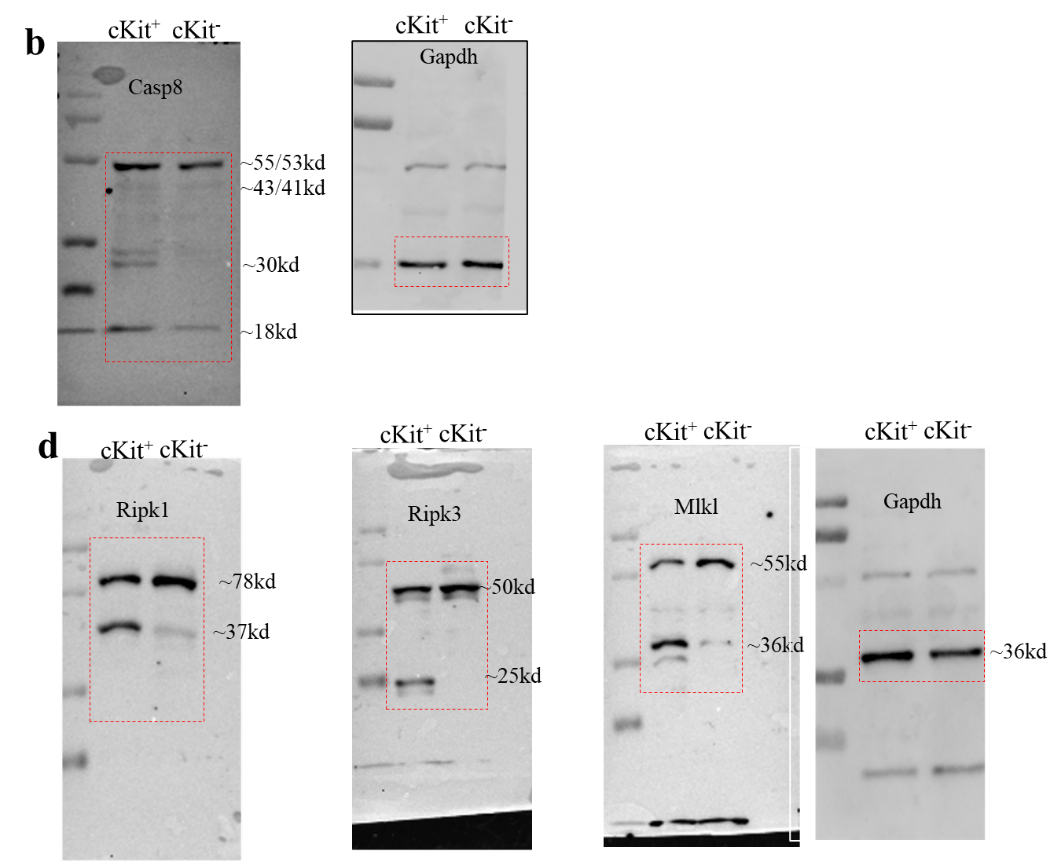


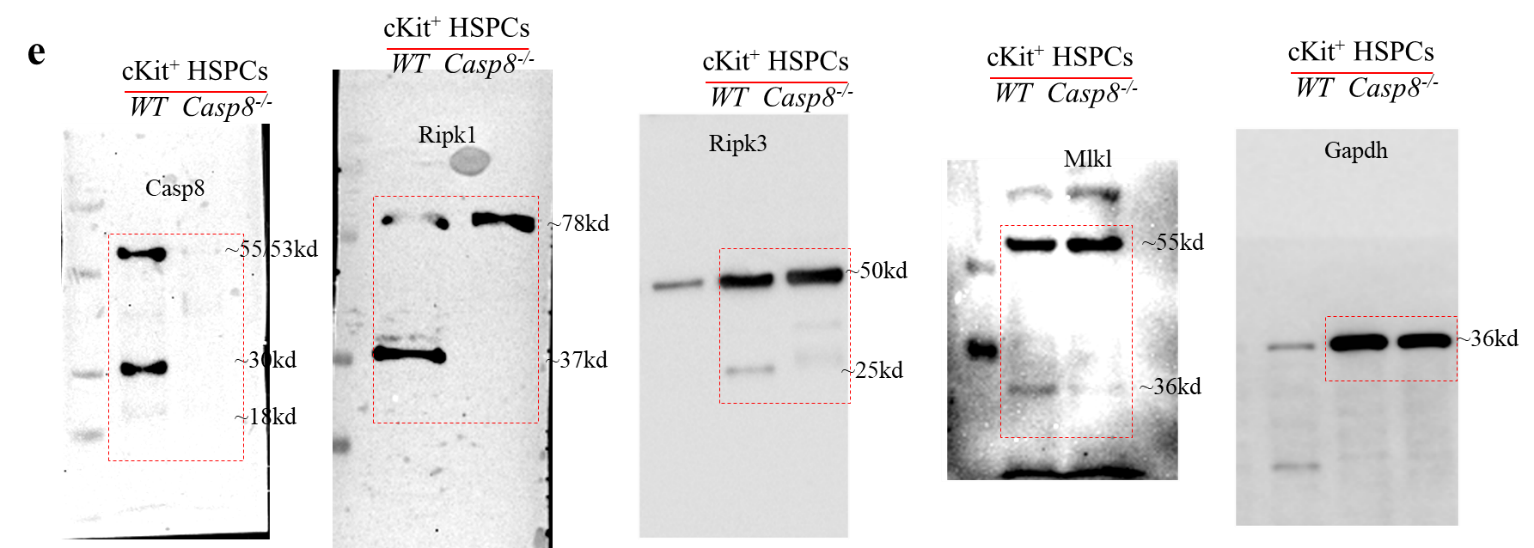


Original western blot data for figure 3


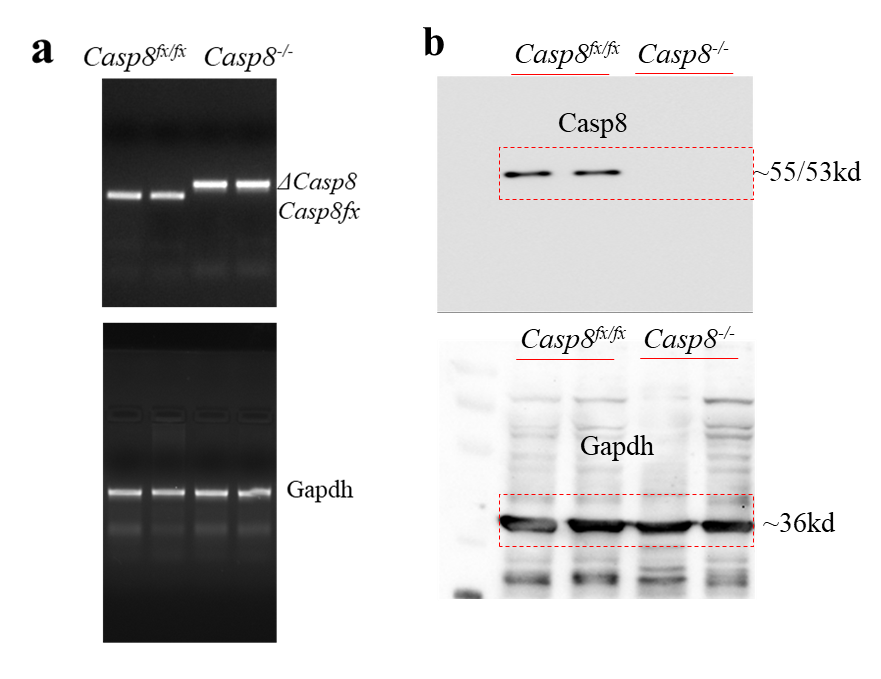


Original western blot data for **figure S1**


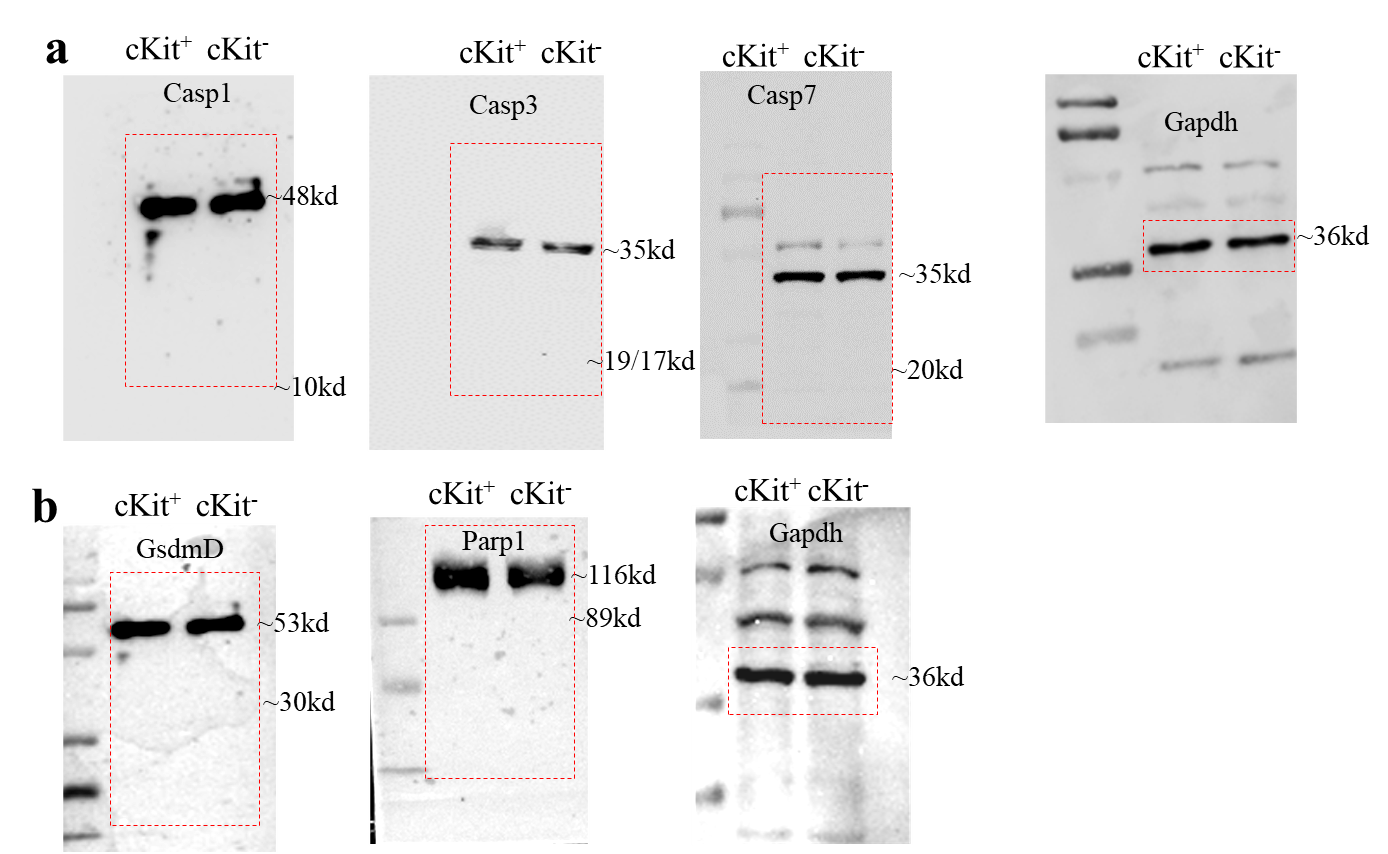

Supplement: Supplementary file 2 — Original Western blot data [file 41419_2024_6660_MOESM2_ESM.docx]
